# Golgiphagy: a novel selective autophagy to the fore

**DOI:** 10.1186/s13578-024-01311-8

**Published:** 2024-10-22

**Authors:** Yifei Chen, Yihui Wu, Xianyan Tian, Genbao Shao, Qiong Lin, Aiqin Sun

**Affiliations:** 1 Institute of Urinary System Diseases, The Affiliated People’s Hospital, Jiangsu University, 8 Dianli Road, Zhenjiang, 212002 China; 2 Department of Basic Medicine, School of Medicine, Jiangsu University, Zhenjiang, Jiangsu 212013 China

**Keywords:** Golgi apparatus, Autophagy, Golgiphagy, Golgi fragmentation, Receptor

## Abstract

The Golgi apparatus is the central hub of the cellular endocrine pathway and plays a crucial role in processing, transporting, and sorting proteins and lipids. Simultaneously, it is a highly dynamic organelle susceptible to degradation or fragmentation under various physiological or pathological conditions, potentially contributing to the development of numerous human diseases. Autophagy serves as a vital pathway for eukaryotes to manage intracellular and extracellular stress and maintain homeostasis by targeting damaged or redundant organelles for removal. Recent research has revealed that autophagy mechanisms can specifically degrade Golgi components, known as Golgiphagy. This review summarizes recent findings on Golgiphagy while also addressing unanswered questions regarding its mechanisms and regulation, aiming to advance our understanding of the role of Golgiphagy in human disease.

## Introduction

The Golgi apparatus was first reported as an “internal reticular apparatus” occupying the perinuclear region by Camillo Golgi in 1898. Its existence was later confirmed by Dalton and Felix using electron microscopy [1]. The Golgi apparatus is a highly polarized organelle [2]. Newly synthesized products from the endoplasmic reticulum (ER) enter the stacks from the cis-side of the Golgi and sequentially pass through various cisternae containing specific enzymes, undergoing post-translational modifications including glycosylation, acetylation, sulphation, phosphorylation, methylation, palmitoylation, and proteolytic cleavage [3], and finally arrive at the trans-Golgi network (TGN), where they are sorted into different vesicles and delivered to specific parts of the cell or secreted outside of it [4]. The classical functions of the Golgi apparatus, membrane transport and glycosylation, are carried out in separate stacks. In vertebrates, however, Golgi stacks collect near the minus end of microtubules, which are aligned by tubular structures and connected laterally to form Golgi ribbon [5]. This process relies on the participation of Golgi matrix proteins and the maintenance of intact microtubule organization [6]. The ribbon structure of the Golgi apparatus allows it to perform a variety of sophisticated functions. For instance, it allows Golgi glycosyltransferases to move laterally between adjacent stacked pools, ensuring precise protein glycosylation [7]. The Golgi ribbon also plays a crucial role in regulating mitotic progression [8], establishing and maintaining cellular polarization, and facilitating directional cell migration [9, 10], among other functions. Additionally, the Golgi is considered to be a central hub for various signaling pathways that regulate cellular processes. It is involved in several biochemical processes including DNA repair [11], stress response [12], control of ionic and reactive oxygen species (ROS) homeostasis [13], apoptosis [14], pro-inflammatory responses [15], and autophagy [16].

**Fig. 1:**
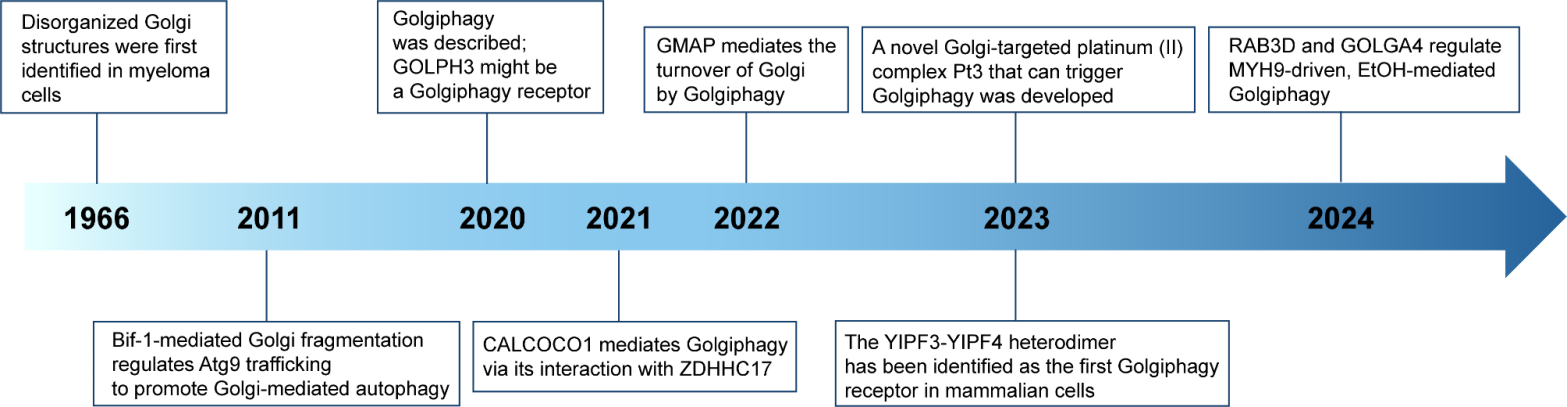
The key discoveries in the Golgiphagy field

The Golgi apparatus is a highly dynamic organelle that is susceptible to fragmentation as a result of various pathological conditions. As early as 1966, disorganized Golgi structures were first identified in myeloma cells [17]. In the following decades, Golgi fragmentation was progressively observed in pathological conditions such as drug stimulation [18], viral infection [19–21], neurodegenerative diseases [22–24], and cancer [25–27]. As research advanced, a potential association between Golgi fragmentation and autophagy was identified. In 2011, Takahashi et al. found that under starvation stress, Bax-interacting factor 1 (Bif-1/Endophillin B1) regulates the transport of Atg9 vesicles from the Golgi apparatus to autophagosomes by mediating Golgi fragmentation and thus promotes autophagosome biogenesis [28]. Subsequently, Gosavi et al. demonstrated that the intact ribbon structure of the Golgi apparatus is the site of mammalian target of rapamycin (mTOR) localization and activation. The researchers discovered that the overexpression of the membrane tether coiled-coil domain containing 88 kDa (GCC88) across the Golgi network resulted in the rupture of the Golgi ribbon, which in turn led to the inhibition of mTOR and a subsequent increase in autophagy levels [29]. Initially, autophagy was thought to maintain cellular homeostasis by non-selectively degrading cytoplasmic components in response to stress. However, more and more studies have demonstrated that cells are capable of exclusively or preferentially degrading specific damaged organelles through selective autophagy. It was the year 2020 when Lu et al. demonstrated that fragmented Golgi is found to accumulate around and be engulfed by autophagosomes during nutrient starvation, thus the concept of Golgiphagy was proposed to describe the process of depletion of fragmented Golgi or Golgi components through selective autophagy [30]. Since then, Golgiphagy has gradually attracted the attention of researchers, and different proteins have been identified to function as Golgiphagy receptors. A new targeted drug against Golgiphagy has also been developed recently (Fig. 1). However, the specific mechanism of Golgiphagy seems to be still being explored.

### Core machinery of autophagy

Autophagy is a highly conserved catabolic process that depends on the lysosomal pathway during the long-term evolution of eukaryotic cells [31]. According to different mechanisms and functions, autophagy is divided into three forms: macroautophagy, microautophagy, and chaperon-mediated autophagy (CMA). Microautophagy is a process whereby specific cytoplasmic components and other intracellular components are directly engulfed by lysosomes for subsequent degradation [32]. CMA is a process by which a cytoplasmic chaperone protein, the heat shock cognate 71-kDa protein (HSC70), binds to a cytoplasmic protein containing a KFERQ or KFERQ-like motif in its amino acid sequence, and then brings this substrate protein to the surface of the lysosome for internalization and rapid lysosomal degradation [33]. Macroautophagy (hereinafter referred to as autophagy) is different from them in the formation of autophagosomes. The key to autophagosome formation in mammals is the initiation of the UNC51-like kinase 1 (ULK1) complex.

In conditions of nutrient starvation, hypoxia, and oxidative stress, the ULK1 complex is activated due to the inactivation of mTOR and the activation of Adenosine 5’-monophosphate-activated protein kinase (AMPK), as well as autophosphorylation of the ULK1 complex. The formation of autophagosomes is facilitated by the recruitment of multiple copies of activated ULK complexes to the sites of autophagosome formation on the ER [34]. The recruitment of ULK1 complexes may involve multiple mechanisms. For example, Vesicle Associated Membrane Protein-associated protein A/B (VAPA/VAPB) could recruit ULK1 complexes through direct interaction with FIP200. GABARAP also binds ULK1 and thus activates and recruits ULK1 complexes to promote autophagy initiation [35].

Additionally, autophagosomes are formed by the contribution of ATG9-containing vesicles derived from TGN. ATG9A is subject to regulation by AMPK- and ULK1-mediated phosphorylation under different conditions, which in turn influences the extent of autophagy [36]. ATG9A can also be recruited to autophagosome forming sites through interactions with ATG13-ATG101, which are part of the ULK1 complex [37].

Subsequently, the class III phosphatidylinositol-3-kinase (PI3KC3) complex I targets the autophagosome formation site in the presence of the ULK1 complex, thereby generating phosphatidylinositol 3-phosphate (PI3P). PI3P can recruit the β-propellers that bind polyphosphoinositides (PROPPIN/WIPI) family, of which WIPI2 is of particular functional importance. The ATG12-ATG5-ATG16L1 complex is recruited to the phagophore membrane by WIPI2B and WIPI2D or by interaction with FIP200, and it can exert E3 enzyme activity to promote the lipidation of mammalian ATG8 family proteins. The ATG8 family proteins may drive phagophore membrane expansion in different ways and play a crucial role in phagophore closure, as well as the subsequent fusion of autophagosomes and lysosomes [34, 35, 38].

Notably, WIPI3 or WIPI4 may interact with ATG2 and recruit it to the phagophore membrane, contributing to lipid transport between the ER and phagophore and promoting phagophore expansion. Meanwhile, ATG2A also interacts with ATG9, phospholipid recombinase vacuole membrane protein 1 (VMP1) and transmembrane protein 41B (TMEM41B) on the ER to form a lipid transfer unit, which maintains equilibrium in the density of phospholipids on each leaflet during lipid transfer [39].

**Fig. 2:**
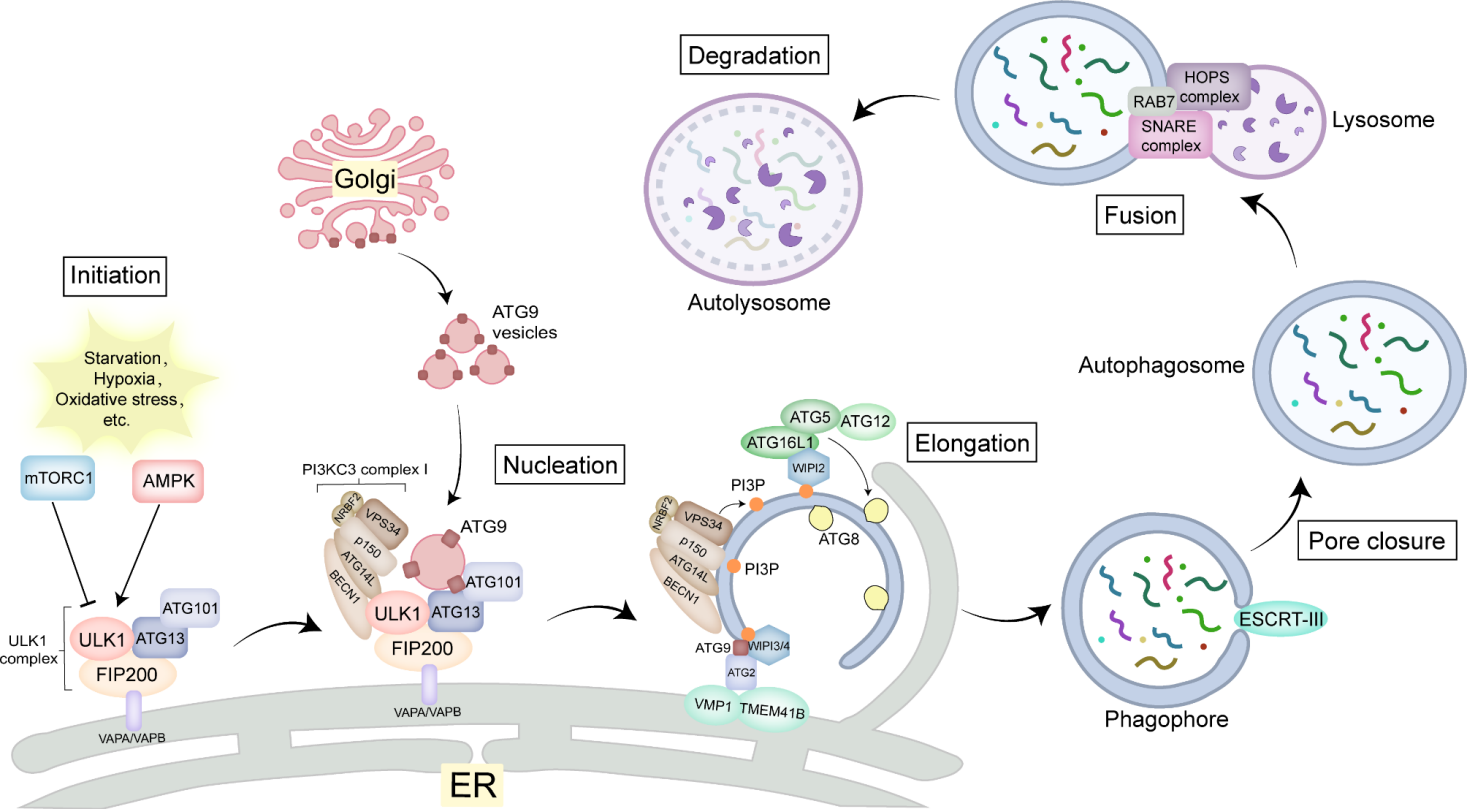
Core machinery of autophagy. In response to various stress conditions, the mTORC1 and AMPK pathways regulate the kinase activity of the ULK1 complex, thereby initiating autophagy. The activated ULK1 complex is recruited near the ER membrane due to the interaction of FIP200 with VAPA/VAPB on the ER. Additionally, ATG9 vesicles are recruited to the ER membrane to provide a membrane source through interaction with the ATG13-ATG101 subcomplex. Subsequently, ULK1 further activates the PI3KC3 complex 1, resulting in the generation of PI3P. WIPI2 then binds to PI3P and further recruits the ATG12-ATG5-ATG16L1 complex to phagophore, thereby mediating ATG8 lipidation. Concurrently, PI3P also recruits WIPI3 or WIPI4 to phagophore, where WIPI3/4 transfer phospholipids from the ER to phagophore through interactions with ATG2, ATG9, as well as VMP1 and TMEM41B on the ER, thereby promoting phagophore expansion. Subsequently, the autophagosome closes through the action of the ESCRT mechanism, after which specific SNARE proteins, HOPS complexes, and small GTPases such as RAB7 mediate the fusion of the autophagosome with the lysosome. Following fusion, the cargo is degraded by hydrolytic enzymes within the lysosome and reused by the cell. Abbreviations: mTORC1, mammalian target of rapamycin complex 1; AMPK, adenosine 5’-monophosphate-activated protein kinase; ULK1, UNC51-like kinase; ER, endoplasmic reticulum; FIP200, focal adhesion kinase family interacting protein of 200 kD; VAPA, VAMP (Vesicle Associated Membrane Protein)-associated protein A; VAPB, VAMP (Vesicle Associated Membrane Protein)-associated protein B; ATG, autophagy-related; PI3KC3, phosphoinositide 3-kinases catalytic subunit type 3; PI3P, phosphatidylinositol 3-phosphate; WIPI, WD repeat structural domain phosphatidylinositol-interacting protein; VMP1, Vacuole membrane protein 1; TMEM41B, Transmembrane Protein 41B; ESCRT, endosomal sorting complex required for transport; SNARE, soluble N-ethylmaleimide-sensitive factor attachment protein receptors; HOPS, homotypic fusion and vacuole protein sorting; RAB, Ras analog in brain

Then, the autophagosome is closed by the action of the endosomal sorting complex required for transport (ESCRT) complex [40, 41]. Subsequently, fusion of autophagosomes and lysosomes occurs mainly in the presence of soluble N-ethylmaleimide-sensitive factor attachment protein receptors (SNAREs) proteins, homotypic fusion and vacuole protein sorting (HOPS) complexes, and small GTPases such as RAB7. Following fusion, the cargo is degraded by hydrolytic enzymes within the lysosome, and the degradation products are then reused by the cell [39, 42] (Fig. 2).

### Involvement of the golgi apparatus in autophagy

**Fig. 3:**
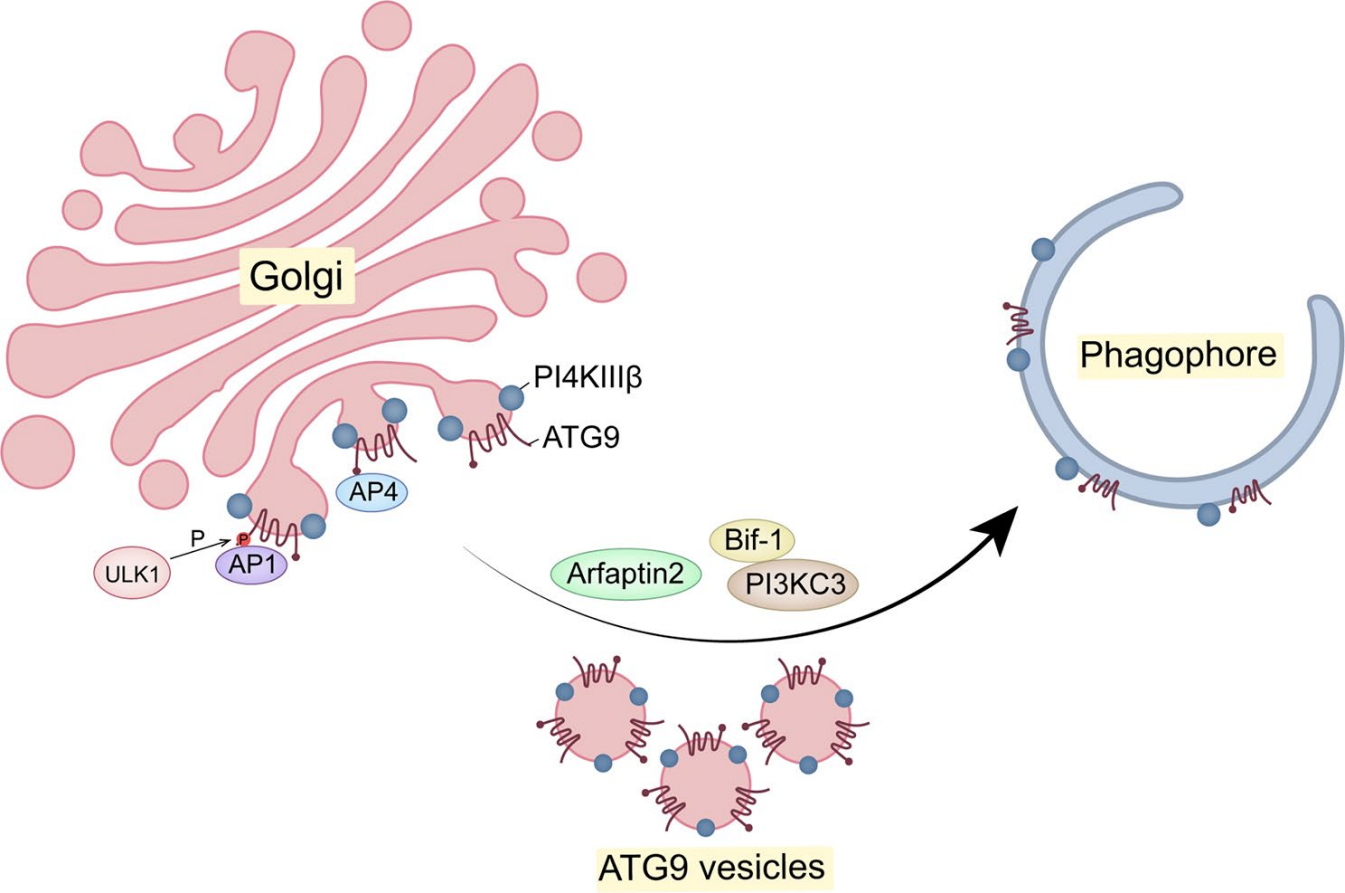
The involvement of Golgi in ATG9 trafficking during autophagy. ATG9 vesicles are transported from the Golgi compartment to autophagosome formation sites (phagophore) via AP1 and/or AP4 complexes, which also deliver PI4KIIIβ. Furthermore, ULK1 phosphorylates ATG9, thereby facilitating ATG9 binding to AP1 complexes. The correct delivery of ATG9 vesicles to phagophore is also regulated by Arfaptin2, Bif-1, and the PI3KC3 complex. Abbreviations: ATG, autophagy-related; AP, adaptor protein; PI4KIIIβ, phosphatidylinositol-4-kinase-IIIβ; ULK1, UNC51-like kinase; Bif-1, BAX-interacting protein 1; PI3KC3, phosphoinositide 3-kinases catalytic subunit type 3

The Golgi, as a central intracellular transport hub, also plays a crucial role in autophagy. Firstly, the Golgi could act as a contributor to the autophagosome membrane to promote autophagosome biogenesis [43, 44]. This may be attributed to the formation and translocation of ATG9 vesicles (Fig. 3). ATG9, the sole multi-transmembrane protein in the ATG family of proteins, is predominantly resides in the membranes of the TGN and endosomes. Geng et al. demonstrated that after autophagy induction two post-Golgi proteins, Sec2 and Sec4, could direct the flow of Golgi-derived Atg9 vesicles to the autophagosome formation site [45]. Also, Yamamoto et al. demonstrated that Golgi-derived Atg9 vesicles were integrated into the outer membrane of autophagosomes [46]. Moreover, the transport of ATG9 vesicles from the Golgi apparatus to the autophagosomes can be regulated by different protein components. For instance, Bif-1, which was previously mentioned, can regulate starvation-induced Golgi membrane fission and the redistribution of Atg9 to the peripheral cytoplasm, which also requires the activity of PI3KC3 [28]. Additionally, ATG9A vesicles from the Golgi can transport active phosphatidylinositol-4-kinase-IIIβ (PI4KIIIβ) to the autophagosome initiation site under the regulation of Arfaptin2, which regulates the production of phosphatidylinositol 4-phosphate (PI4P) on the phagosome membrane and thus promotes autophagy [47]. In response to starvation stress, ULK1 phosphorylates ATG9A to promote the interaction of ATG9A with the adaptor protein 1 (AP1) complex, which in turn promotes the movement of ATG9A from the TGN to the ATG9A compartment and facilitates the initiation of autophagy [48]. Whereas, when ATG9A export from the TGN is impeded due to AP4 deletion, the delivery of ATG9A vesicles to autophagosomes is diminished, thereby impairing autophagosome formation [49, 50].

In addition, certain Golgi-related proteins are involved in regulating the process of autophagy. For example, the Golgi Re Assembly Stacking Protein 55 (GRASP55) de-O-GlcNAcylates upon glucose starvation and targets the autophagosome-lysosome interface through interactions with LC3-II and lysosomal associated membrane protein 2 (LAMP2), where it serves as a bridge to promote autophagosome-lysosome fusion [51]. GRASP55 also further promotes autophagosome maturation by facilitating the assembly of the PI3K UVRAG complex [52]. In response to autophagic stimuli, RAB2 dissociates from the Golgi and promotes phagophore formation by recruiting and activating the ULK1 complex. Subsequently, RAB2 shifts to interact with rubicon like autophagy enhancer (RUBCNL) and Syntaxin 17 (STX17) to further recruit HOPS complexes into autophagosomes to promote fusion with lysosomes [53]. Furthermore, Coat Protein Complex I (COP I) vesicles, which are involved in retrograde transport of proteins from the Golgi to the ER, have also been shown to play a role in autophagy. It has been shown that deletion of the COP I subunit leads to disruption of Golgi structure and accumulation of autolysosome-like structures in plant cells, which inhibit autophagy [54]. A recent study demonstrated that COP I vesicles function upstream of mTORC1 and activate autophagy by regulating the phosphorylation of S6 Kinase 1 (S6K1), which in turn plays a key role in the formation of autophagosomes during mineralization [55].

### Golgiphagy receptors

The Golgi apparatus is not only involved in the regulation of autophagy, but can itself be selectively degraded as an autophagic cargo. In recent years, research has identified several proteins that may act as Golgiphagy receptors to mediate the degradation of Golgi components through the autophagic pathway. These include Golgi phosphoprotein 3 (GOLPH3), calcium binding and coiled-coil domain protein 1 (CALCOCO1), Golgi microtubule-associated protein (GMAP), as well as members 3 and 4 of the Yip1 domain family (YIPF3 and YIPF4). The following provides a detailed description of the Golgiphagy receptors that have been studied in recent years.

#### GOLPH3

**Fig. 4:**
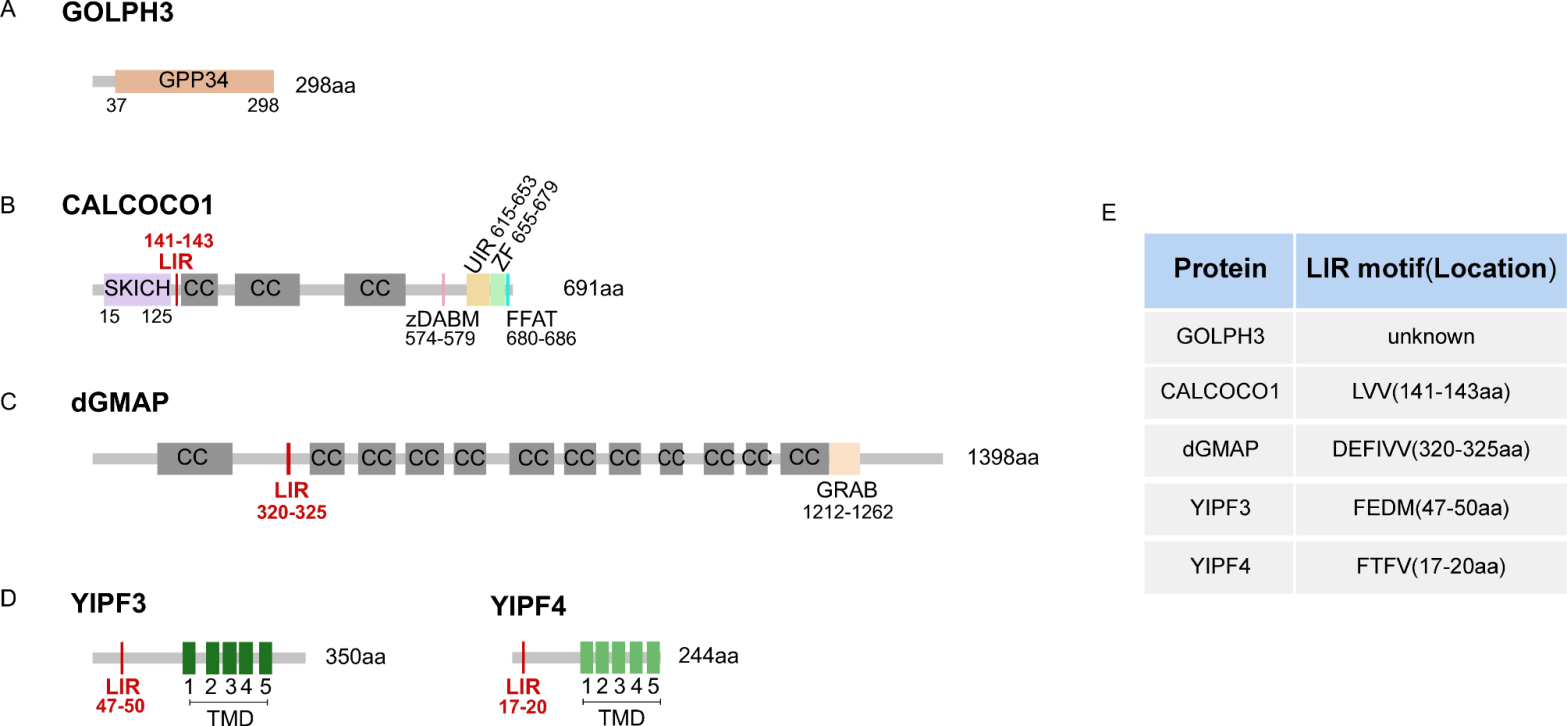
The structure of the Golgiphagy receptors. (**A**) Schematic structure of GOLPH3 protein. GPP34, PI4P-binding domain. (**B**) Schematic structure of CALCOCO1 protein. SKICH, SKIP carboxyl homology domain; LIR, LC3-interacting region; CC, coil-coil region; zDABM, zDHHC-AR-binding motif; UIR, UDS-interacting region; ZF, Zinc Finger; FFAT, two phenylalanines in an acidic tract domain. (**C**) Schematic structure of dGMAP protein. GRAB, GRIP-related Arf-binding domain. (**D**) Schematic structures of the YIPF3 protein and the YIPF4 protein. TMD, transmembrane domain. (**E**) A table of the amino acid sequences as well as the positions of the LIR motifs in these Golgiphagy receptors

GOLPH3 is also referred to GMx33, GPP34, or MIDAS, and its yeast homologue is Vps74p. GOLPH3 is a highly conserved protein initially identified in a proteomic characterization of the rat Golgi apparatus. It is subsequently recognized as a Golgi matrix protein, which is mainly enriched at the trans-side of the Golgi apparatus through the conserved C-terminal domain dubbed GPP34 [56, 57](Fig. 4A). GOLPH3 has multiple functional roles in cells. GOLPH3 can be rapidly exchanged between the cytoplasmic and Golgi-associated pools. Additionally, it has been found to be associated with tubules and vesicles that leave the Golgi apparatus [58]. Dippold et al. discovered that GOLPH3 is crucial in anterograde trafficking from the Golgi apparatus to the plasma membrane. Furthermore, GOLPH3 can specifically attach to the Golgi membrane by binding to PI4P and myosin XVIIIA (MYO18A), thus maintaining the flat appearance of the trans-Golgi [59]. GOLPH3 also maintains the localization of specific glycosyltransferases within the Golgi apparatus [60–62].

**Fig. 5:**
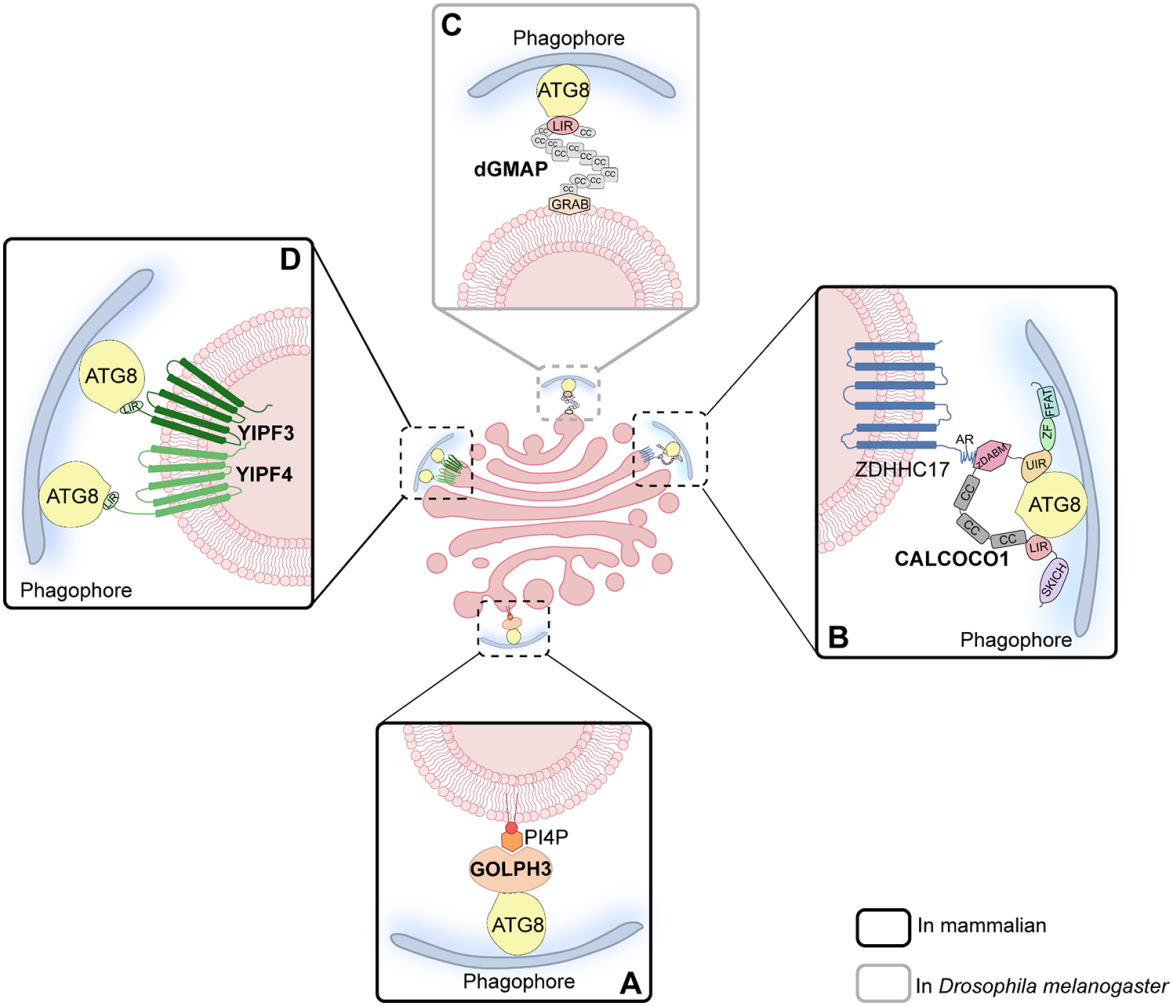
Models of Golgiphagy receptors-mediated Golgiphagy upon nutrient starvation in mammalian or *Drosophila melanogaster*. (**A**) GOLPH3, which is located on the trans-Golgi by binding PI4P, interacts with ATG8 to promote the encapsulation of Golgi fragments by phagophore. (**B**) CALCOCO1 localizes to the Golgi apparatus through its zDABM motif binding to the AR domain of ZDHHC17. Subsequently, CALCOCO1 interacts with ATG8 through its LIR and UIR motifs, recruiting the autophagy machinery and thus facilitating the degradation of Golgi fragments. (**C**) In *Drosophila melanogaster*, dGMAP can directly bind to ATG8 through the N-terminal LIR motif, thereby mediating the autophagy pathway degradation of the Golgi fragments. (**D**) YIPF3 and YIPF4, which are anchored to the Golgi through 5 tightly stacked transmembrane domains, form a heterodimer. As the only membrane-embedded Golgiphagy receptors, they interact with ATG8 to mediate Golgiphagy. Abbreviations: GOLPH3, Golgi phosphoprotein 3; CALCOCO1, calcium binding and coiled-coil domain protein 1; dGMAP, Golgi microtubule-associated protein in *Drosophila melanogaster*; YIPF3, the member 3 of Yip1 domain family; YIPF4, the member 4 of Yip1 domain family; PI4P, phosphatidylinositol 4-phosphate; AR, ankyrin repeat; SKICH, SKIP carboxyl homology; LIR, LC3-interacting region; CC, coil‐coil regions; zDABM, zDHHC-AR-binding motif; UIR, UDS‐interacting region; ZF, Zinc Finger; FFAT, two phenylalanines in an acidic tract

Lu et al. demonstrated that GOLPH3 may act as a cargo receptor to target the Golgi apparatus to autophagosomes for lysosomal degradation [30] (Fig. 5A). First, their study revealed that under conditions of starvation, treatment with various Golgi stress inducers, such as Brefeldin A, Nocodazole, Monensin, and Nigericin, resulted in increased co-localization of GM130-RFP (a cis-Golgi marker) and TGN46-RFP (a trans-Golgi marker) with LC3B-GFP (an autophagosome marker). Furthermore, transmission electron microscopy revealed that starvation treatment results in the accumulation of Golgi fragments around autophagosomes and phagocytosis by autophagosomes, supporting the occurrence of Golgiphagy and suggesting that Golgi stress inducers may promote Golgiphagy. Additionally, it was discovered that endogenous GOLPH3 in H9c2 cells, HUVECs, and HA-VSMCs cells can interact with LC3B under normal, starvation, or hypoxia-stimulated conditions. Moreover, the knockdown of GOLPH3 resulted in a decrease in the co-localization of Golgi marker proteins with LC3B in H9c2 cells, HUVECs, and HA-VSMCs. This led to the speculation that GOLPH3 may function as a Golgiphagy receptor. However, further investigation is required to elucidate the molecular mechanism underlying GOLPH3’s function as a Golgiphagy receptor.

#### CALCOCO1

CALCOCO1 is a paralogous homologue of two previously described autophagy receptor proteins, CALCOCO2/NDP52 and CALCOCO3/TAX1BP1. These three proteins constitute the CALCOCO family, sharing the same conserved domains: an N-terminal SKIP carboxyl homology (SKICH) domain, middle coil-coil regions (CC), and an atypical LC3-interacting region (LIR) motif [63]. Furthermore, CALCOCO1 contains a UDS-interacting region (UIR) that functions in conjunction with the LIR to bind LC3 [64](Fig. 4B). Previous research has demonstrated that CALCOCO1 is involved in transcriptional co-activation, glucose metabolism, and calcium signaling. Stefely et al. later expanded on the role of CALCOCO1 in mTOR-regulated selective autophagy, discovering through mass spectrometry proteomic analysis that CALCOCO1 may be a novel autophagy-associated protein. The authors also demonstrated that CALCOCO1 can physically interact with LC3. In addition, the deletion of the CALCOCO1 gene has been shown to disrupt ER-phagy [65]. Nthiga et al. demonstrated that CALCOCO1 is capable of mediating ER-phagy. CALCOCO1 interacts with VAPA and VAPB proteins in the ER through a novel FFAT-like motif and with the ATG8 family proteins through LIR and UIR motifs. This interaction recruits autophagic machinery to degrade cargo [64].

Recently, Nthiga et al. discovered that CALCOCO1 also regulates Golgi size and morphology by mediating Golgiphagy in eukaryotic cells through its interaction with the zinc finger DHHC-type palmitoyltransferase 17 (ZDHHC17) [66] (Fig. 5B). Under basal conditions, CALCOCO1 can interact with ZDHHC17 and ZDHHC13 located on the Golgi apparatus and thus anchored to it. This interaction is mediated by the zDHHC-AR-binding motif (zDABM) on CALCOCO1 with the ZDHHC17 N-terminal ankyrin repeat (AR) domains. Studies show that CALCOCO1 can recruit most of the ZDHHC17-containing Golgi fragments produced by starvation induction into autophagosomes and deliver them to lysosomes for degradation by interacting with LC3/GABARAP proteins. The authors demonstrated that the absence of interaction between CALCOCO1 and ZDHHC17, or the presence of a mutant CALCOCO1 lacking LIR and UIR motifs, leads to a reduction or impairment in the autophagic degradation of Golgi components. It is noteworthy that CALCOCO1-mediated Golgiphagy is induced by the need to remove excess Golgi components produced during stress in order to restore the pre-stress state of the Golgi apparatus. Under nutrient-sufficient conditions, constitutive Golgi turnover may not necessitate CALCOCO1-ZDHHC17-dependent degradation. Alternatively, it was discovered that TAX1BP1, which shares significant sequence similarity and identity with CALCOCO1, could also mediate its interaction with ZDHHC17 through the AR-zDABM interface. Therefore, it is worth investigating whether TAX1BP1 also functions in a similar mode in Golgiphagy.

#### GMAP

Human GMAP-210 (Golgi microtubule-associated protein 210) is a 210 kDa peripheral Golgi protein located in the cis-Golgi network (CGN), classified as a member of the golgin family of proteins [67]. hGMAP-210, which plays a role in maintaining the structural integrity of the Golgi apparatus, binds to the Golgi apparatus through its NH_2_ terminus and interacts directly with microtubules through its COOH-terminal domain [68]. hGMAP-210 acts at the crossroads between the anterograde and retrograde transport, connecting the ER to the Golgi apparatus. The overexpression of hGMAP-210 can block both anterograde and retrograde transport between the ER and the Golgi apparatus, and significantly alter the morphology of the Golgi complex, resulting in the accumulation of vesicles to form large clusters [69]. In contrast to previous studies, Sato et al. demonstrated that the knockdown of hGMAP-210 results in the Golgi apparatus breakage and significant densification. This may be due to differences in knockdown efficiency. Additionally, they found that hGMAP-210 acts as a Golgi vesicle tether in vivo [70]. Research has demonstrated that hGMAP-210 plays a crucial role in maintaining the Golgi band around the centrosome through its interactions with the Golgi membrane and γ-tubulin [71]. Additionally, hGMAP-210 is necessary for the efficient glycosylation and cellular translocation of a wide variety of proteins [72]. The ortholog of hGMAP-210 was identified in *Drosophila melanogaster*, referred to as dGMAP by Friggi-Grelin et al., and was found to be localized at the cis-side of the Golgi apparatus through the GRAB (GRIP-related Arf-binding) domain of the COOH-terminus (Fig. 4C). They showed that the overexpression of dGMAP resulted in a disruption of the Golgi stacks and a significant inhibition of translocation to the plasma membrane. Conversely, the knockdown of dGMAP did not cause any structural changes in the Golgi apparatus, and cis transport seemed to be unaffected [73].

Rahman et al. demonstrated that dGMAP directly binds to Atg8a, regulating Golgi transitions and controlling the size and morphology of the Golgi complex [74](Fig. 5C). In *Drosophila*, there are only two Atg8 proteins: Atg8a and Atg8b, of which Atg8b is expressed exclusively in the male germline and required for male fertility. Sequence analysis reveals that Atg8a and Atg8b are almost identical, both containing LDS sites [75]. In this study, the authors used CRISPR to generate the Atg8a^K48A/Y49A^ (Atg8a LDS) mutant. They discovered that dGMAP was significantly upregulated in Atg8a^K48A/Y49A^ files through quantitative proteomics analysis. They then used immunofluorescence confocal microscopy to examine the expression pattern of dGMAP in the adult *Drosophila* brain, revealing a significant increase in the number and size of dGMAP puncta in the adult brain of Atg8a^K48A/Y49A^ mutant flies. Furthermore, the authors found that the Atg8a^K48A/Y49A^ mutation did not impair autophagy. They also observed an accumulation of the Golgi marker GM130 in Atg8a^K48A/Y49A^ mutants. These findings suggest that selective autophagy regulates the size and morphology of the Golgi apparatus, as well as its turnover. Additionally, they demonstrated the significance of the LIR motif at positions 320–325 of dGMAP for dGMAP-Atg8a interaction (Fig. 4C). Thus, a dGMAP LIR mutant (dGMAP^F322A/V325A^) was generated using CRISPR-Cas9 technology. In the dGMAP^F322A/V325A^ mutant files, GM130 accumulated significantly, and a clear increase in the area and length of its Golgi compartment could be observed using transmission electron microscopy. These results suggest that in *Drosophila*, dGMAP may act as a Golgiphagy receptor to regulate Golgi turnover and control Golgi size and morphology by binding to Atg8a.

#### YIPF3 and YIPF4

Hickey et al. recently identified the only currently available membrane-embedded Golgiphagy receptors, YIPF3 and YIPF4 [76](Fig. 5D). These proteins are homologues of yeast Yip1p and Yif1p, which have been shown to play important roles in ER to Golgi transport [77]. YIPF3 and YIPF4 are members of the YIP family (YIPF). The YIPF family exhibits strong structural similarities. They possess a soluble N-terminal domain, which is 100–150 residues in length and faces the cytosol. Additionally, they have 5 tightly stacked transmembrane domains (TMDs) and a C-terminal hydrophobic domain oriented into the lumen of the endomembrane system [78](Fig. 4D). Research has demonstrated that YIPF3 and YIPF4 are primarily located in the cis-Golgi, and that YIPF3 and YIPF4 may recycle within the Golgi apparatus and to some extent between the ER and the ERGIC. Tanimoto et al. have also shown that YIPF4 appears to form a complex with the Golgi form of YIPF3, and that knockdown of YIPF4 results in a reduction of YIPF3. Additionally, the knockdown of either YIPF3 or YIPF4 resulted in varying degrees of Golgi breakage. The depletion of YIPF4 also caused fragments to be displaced from the proximal nuclear region. However, the Golgi apparatus functioned normally after knocking down YIPF3 or YIPF4 in HeLa cells. This suggests that YIPF3 or YIPF4 may not be essential regulators of secretion [78, 79].

To reveal the selectivity of autophagy during nutrient stress, Hickey et al. treated wild-type and autophagy-deficient cells with starvation or amino acid deprivation, and then identified large amounts of proteins, termed candidate autophagy proteins (CAPs), by complementary proteomic analysis. The study found that ER and Golgi proteins were significantly more abundant in CAPs. Additionally, CAPs showed a strong enrichment of Golgi membrane proteins compared to peripheral Golgi-associated proteins. Thus, the authors again used proteomics approaches to identify the Golgi transmembrane proteins YIPF3 and YIPF4 as Golgiphagy receptor candidates. The researchers found that YIPF4 can directly interact strongly with GABARAPL2 through complementary proximity biotinylation assays. Additionally, the authors proved that the degradation of YIPF3 and YIPF4 during starvation is dependent on GABARAPs rather than LC3s. Also, they found that Keima-YIPF3 and Keima-YIPF4 fluxes were increased under nutrient stress in a FIP200-/- dependent manner by flow cytometry measurements, indicating that YIPF3 and YIPF4 can be degraded by autophagy. Then, within three hours of starvation, the authors observed large amounts of YIPF4 and YIPF3, as well as the co-localization of YIPF4 with LAMP1 and LC3B using fluorescence microscopy, suggesting that autophagosomes capture YIPF3 and YIPF4 from the Golgi and translocate them to the lysosome. Subsequently, the authors knocked out YIPF4 and found that this specifically disrupted the degradation of Golgi membrane proteins. In addition, they noted that deletion of YIPF3 and YIPF4 had essentially no effect on the number of ATG9 vesicles or Golgi morphology. This suggests that autophagosome biogenesis is not impaired in the absence of YIPF3 and YIPF4.

Finally, their previous study showed that in vitro human embryonic stem cell differentiation towards induced neurons is closely related to autophagy-dependent ER and Golgi proteome remodeling [80]. In this study, the authors found that neurons lacking YIPF4 exhibited a selective accumulation of Golgi membrane proteins to a similar extent as autophagy-deficient neurons, with a pattern of accumulation similar to that of the CAPs produced by nutritional stress. Thus, YIPF3 and YIPF4 play roles as Golgiphagy receptors not only during nutrient starvation but also during cell differentiation.

#### CLC2

A recent study by Zhou et al. has shown that CLATHRIN LIGHT CHAIN 2 (CLC2) can interact with ATG8 to promote Golgi recovery after heat stress (HS) in plants [81]. The study found that short-term acute HS induced vacuolization of plant Golgi and inhibited Golgi-mediated membrane transport. At this time, ATG8 puncta in plant cells are formed under the action of ATG conjugation system (ATG5, ATG7 and ATG16), and then localized to the Golgi stacks with swollen cisternae. It is crucial to acknowledge that the formation and targeting of ATG8 to the disrupted Golgi apparatus in HS-treated plant cells is not dependent on the classical autophagy pathway, and that the ATG8-positive vesicles observed are not autophagosomes in the traditional sense at this time. Then, the authors utilized TurboID, a protein proximity labeling method, to identify CLC2 as an ATG8-interacting partner. CLC2 interacts with ATG8 through the AIM-LDS interface and is recruited near the bud-like structures derived from the vesicular Golgi. This interaction mediates subsequent vesicle budding and fusion of these vesicles with vacuole membranes, promoting Golgi recovery.

In plants, CLC2 is not a strict Golgiphagy receptor, but rather acts as a regulator that can mediate the recovery of the Golgi from HS with the involvement of ATG8. It is worthy of consideration whether the mammalian homologue of CLC2 plays a similar role or acts as a bona fide Golgiphagy receptor mediating Golgi turnover, which would be a worthwhile area for further investigation.

### Regulation of Golgiphagy

**Fig. 6:**
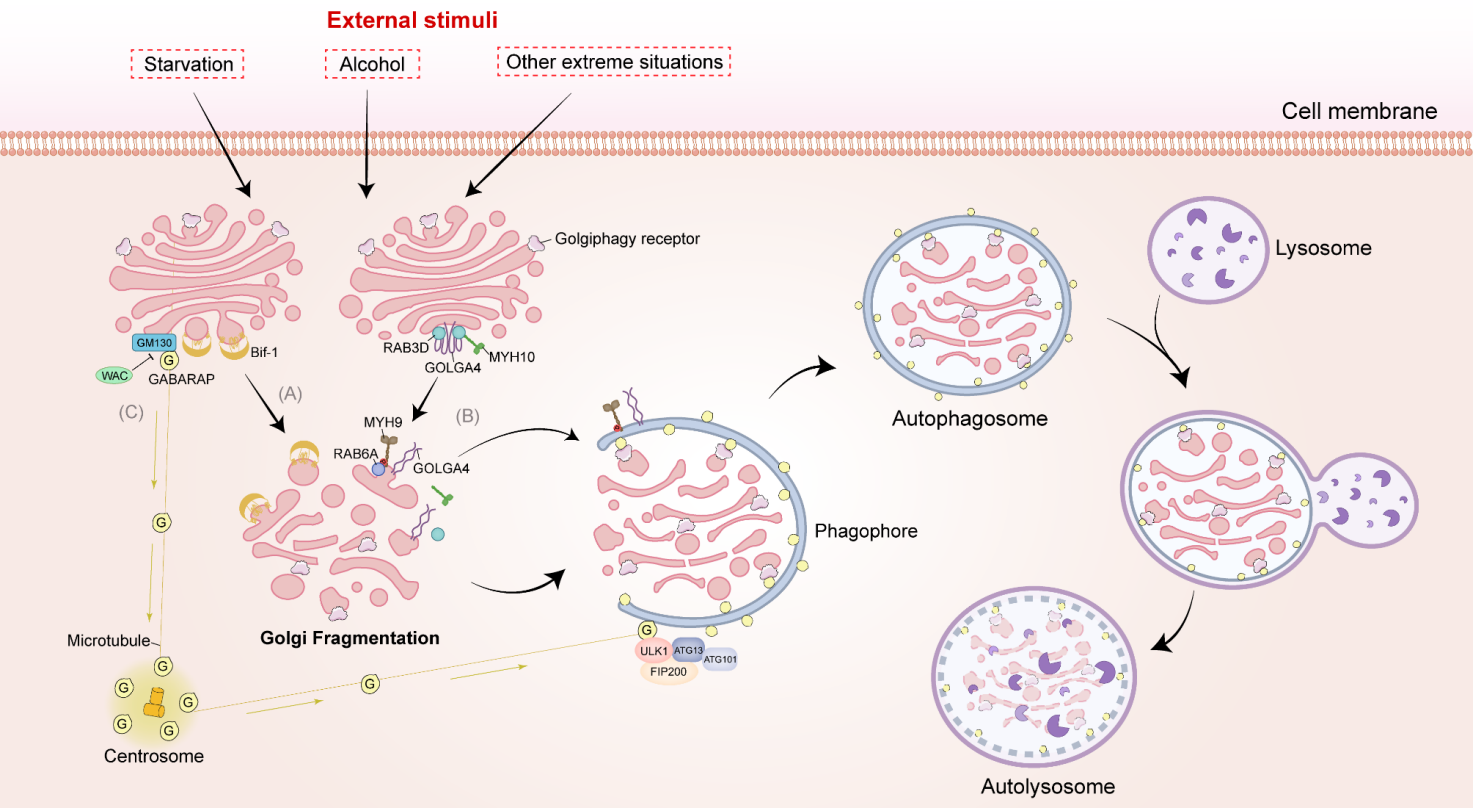
The process and regulation of Golgiphagy. Under various external stimuli, including starvation, alcohol exposure and other extreme situations, the Golgi apparatus undergoes fragmentation, which in turn triggers the process of Golgiphagy. The Golgiphagy receptors, localized on the Golgi, mediate phagophore wrapping around the fragmented Golgi by binding to ATG8 on the phagophore. The phagophore continue to extend and then close, forming autophagosomes which in turn fuse with lysosomes thereby degrading Golgi components. The process of Golgiphagy is subject to regulation by several factors. (**A**) Bif-1 could rupture the Golgi membrane during starvation, thereby inducing Golgiphagy. (**B**) RAB3D, MYH10, and GOLGA4 form a complex to maintain the structural integrity of the Golgi apparatus. Upon ethanol treatment, RAB3D is reduced, MYH10 separates from the Golgi, and MYH9 exerts force to disperse the Golgi membrane by binding to it via RAB6A. Concurrently, the conformation of the GOLGA4 protein is altered to facilitate the formation of the phagophore from the fragmented Golgi cisterna. (**C**) In conditions of starvation, WAC inhibits the binding of GABARAP to GM130, thereby allowing the maintenance of the centrosomal GABARAP pool. The centrosomal GABARAP is transported along microtubules to the phagophore, where it mediates the autophagic activation of the ULK1 complex and promotes the biogenesis of autophagosomes during Golgiphagy. Abbreviations: ATG, autophagy-related; Bif-1, BAX-interacting protein 1; RAB, Ras analog in brain; MYH10/NMIIB, non-muscle myosin II B; MYH9/NMIIA, non-muscle myosin II A; GOLGA4, golgin A4; WAC, WW domain-containing adaptor with coiled coil; GM130/GOLGA2, Golgin subfamily A member 2; GABARAP, GABA Type A Receptor-Associated Protein; ULK1, UNC51-like kinase

Various stress conditions, such as nutrient starvation, growth factor deprivation, hypoxia or oxidative stress, ER stress, and pathogen infection, can stimulate the induction of autophagy [82]. It was found that under starvation conditions, the membrane curvature-driving protein Bif-1 is able to localize to the Golgi membrane via the amphiphilic fragment helix 0 (H0) of its N-BAR structural domain and promote membrane curvature via helix 1 insert (H1I), which in turn induces tabularization and fracture of the Golgi membrane [28](Fig. 6A). The available evidence suggests that these Golgi bodies, which undergo fragmentation due to nutrient starvation, accumulate around autophagosomes and are then engulfed into autophagosomes. This phenomenon has also been observed under some other stress conditions, including hypoxia, general autophagy inducers, and Golgi stress inducer treatments [30]. Moreover, Hickey et al. employed quantitative proteomics to demonstrate that the majority of the reduction in Golgi membrane protein levels observed under nutrient stress was attributable to their degradation by selective autophagy. They also proposed that Golgiphagy plays a pivotal role in cellular adaptation to nutrient stress [76].

Additionally, chronic ethanol treatment has been demonstrated to result in the dispersion of Golgi membranes in hepatocytes. This is due to the fact that ethanol treatment leads to the phosphorylation of S1943, the heavy chain of non-muscle myosin II A (NMIIA/MYH9), which thereby binds to the RAB6A GTPase on the Golgi membrane, forcing to the Golgi to disintegrate and rupture [83]. And A. J. MACK et al. elucidated that these ethanol-induced dispersed Golgi membranes can serve as a source of phagophore membranes in the initiation phase of Golgiphagy, which mediate the onset of Golgiphagy and promote the cellular stress response to alcohol exposure [84].

Currently, Golgiphagy has not been well studied, but are these the only factors that can trigger Golgiphagy? The Golgi apparatus is known to be a highly dynamic organelle, susceptible to perturbations in its morphology and function by different stimuli. For instance, in response to DNA damage, the DNA damage protein kinase DNA-PK phosphorylates GOLPH3, leading to an increased interaction with MYO18A, which abnormally increases the tension of Golgi fragmentation [11]. In HCV infection, immune-related guanosine triphosphatase M protein (IRGM) can lead to Golgi fragmentation by regulating AMPKα and Golgi brefeldin A resistant guanine nucleotide exchange factor 1 (GBF1), a guanosine nucleotide exchange factor (GEF) for Arf-GTPases [85]. Bacterial infections such as Streptococcus pneumoniae, Rickettsia, and Streptococcus pyogenes have also been shown to disrupt the Golgi structure [86–88]. Beyond that, as of now, there are still many triggers for Golgiphagy are undiscovered, and therefore, need to be characterized.

Golgiphagy triggered by these stress conditions is regulated by multiple protein molecules (Fig. 6). Firstly, the role of Golgiphagy receptors and ATG8 family proteins is of paramount importance. During nutrient starvation, receptors that are initially anchored to the Golgi apparatus, such as GOLPH3, GAMP, YIPF3-YIPF4, or those that are localized to the Golgi apparatus through binding to specific Golgi proteins, such as CALCOCO1, can facilitate the recruitment of starvation-induced Golgi fragments into the autophagosome through direct interactions with LC3/GABARAP, which plays a crucial role in mediating Golgiphagy.

Furthermore, under normal conditions, non-muscle myosin II B (NMIIB/MYH10) forms a complex with the Golgi matrix protein golgin A4 (GOLGA4) via the RAB3D GTPase in trans-Golgi. Whereas chronic ethanol treatment has been observed to result in a reduction in RAB3D levels, the disintegration of the complex, MYH10 dissociation from the Golgi, and MYH9 binding to the Golgi membrane, which ultimately leads to the dispersion of the Golgi membrane. Concurrently, the down-regulation of RAB3D alters the protein conformation of GOLGA4 on the Golgi membrane, transforming it from a curved to an extended form. This exposes the N-terminal end of GOLGA4 to the cytoplasm, promoting the formation of phagophore from the Golgi membrane (Fig. 6B). It facilitates the occurrence of intracellular Golgiphagy in response to alcohol exposure [84].

In addition, the Golgi-centrosomal GABARAP pool controlled by WW domain-containing adaptor with coiled coil (WAC) and Golgin subfamily A member 2 (GOLGA2/GM130) may play an important role in the initiation of Golgiphagy (Fig. 6C). Under basal conditions, GM130 tethers GABARAP to the Golgi to maintain the normal structure and function of the Golgi. Under conditions of nutrient starvation, WAC is phosphorylated, which in turn replaces GABARAP for binding to GM130. GABARAP then dissociates from the Golgi and moves through microtubules to the pericentriolar material of the centrosome, maintaining a nonlipidated centrosomal GABARAP pool. Consequently, the nonlipidated form of GABARAP facilitates ULK1 activation by directly interacting with ULK1 via the LIR motif, which may be involved in the formation of autophagosomes during the pre-Golgiphagy phase. The recruitment of the GABARAP-ULK1 complex to phagophore surrounding the Golgi may be attributed to the binding of ATG16L and FIP200 in the ULK1 complex [89].

It is notable that existing studies have observed an increased localization of WIPI2 on the autophagic precursor Golgi membranes in response to stress conditions that can trigger Golgiphagy, such as starvation or alcohol exposure. The recruitment of WIPI2 to the membrane of autophagic precursors is facilitated by its binding to the RAB11A GTPase, as well as to PI3P [90]. This is followed by the recruitment of ATG12-ATG5-ATG16L1 complex to the WIPI2, which promotes LC3 lipidation and thus Golgiphagy [91]. However, further research is required to elucidate the precise mechanism by which WIPI2 is targeted to the Golgi membrane.

At present, the field of Golgiphagy is still in its nascent stages, suggesting that there are numerous additional protein regulators of this process yet to be identified.

### Insights into human diseases related to golgiphagy

Golgiphagy regulates structural abnormalities in the Golgi by degrading Golgi fragments to maintain cellular homeostasis. Based on the complexity and significance of Golgi function, Golgiphagy may be associated with various human diseases, including neurodegenerative diseases and cancer.

### Neurodegenerative diseases

Neurodegenerative diseases (NDDs) are a group of neurological disorders characterized by the progressive loss of neurons in the central nervous system (CNS) or peripheral nervous system (PNS) [92]. Golgi fragmentation has been shown to be associated with a variety of neurodegenerative diseases.

Alzheimer’s disease (AD) is an age-related neurodegenerative disease of the CNS characterized by progressive dementia and cognitive deficits. The pathological hallmark of AD is the formation of extracellular amyloid plaques formed by secreted amyloid β-protein(Aβ) peptides and neurofibrillary tangles(NFTs) caused by hyperphosphorylated tau protein deposits [93]. Aβ is produced by the proteolytic cleavage of amyloid precursor protein (APP) by β- and γ-secretase enzymes [94]. Several studies have shown that altered Golgi morphology can be found in neurons of AD patients [23, 95]. Joshi et al. demonstrated that the Golgi apparatus is fragmented in both AD cell cultures and mouse models, using tissue culture cells and APPswe/PS1E9 transgenic mice. They showed that this could be due to the activation of cell cycle protein-dependent kinase 5 (Cdk5) and the subsequent phosphorylation of GRASP65 by Aβ accumulation, leading to Golgi fragmentation triggered by GRASP65 dysfunction. Subsequently, Golgi fragmentation in turn accelerated APP trafficking and increased Aβ production [96]. Based on this, the researchers suggest that Golgi fragmentation in AD may enhance Aβ production and hyperaccumulation by accelerating APP trafficking and amyloidogenic processing of the β-secretase BACE1 and the γ-secretase progerin 1 (PS1), thereby contributing to the development of AD [97]. In addition, Golgi fragmentation triggers tau hyperphosphorylation, which also accelerates the pathogenesis of AD [98].

Parkinson’s disease (PD) is the second most common neurodegenerative disease after AD [99]. The pathology of PD is characterized by the loss of dopamine-containing neurons and the formation of intracellular protein aggregates known as Lewy bodies, of which α-synuclein is considered to be the main component [100]. Golgi fragmentation has been observed in PD [101–103]. Golgi fragmentation in dopamine neurons may be caused by the disruption of ER to Golgi transport due to the aggregation of α-synuclein [104]. However, subsequent research has shown that Golgi fragmentation is caused by changes in the homeostasis of specific Rab and SNARE proteins that precede and may even trigger α-synuclein aggregation and inclusions formation, as well as alterations in anterograde and retrograde transport between the ER and the Golgi complex [105]. In a model of dopaminergic neuronal degeneration, centrosome aggregation and Golgi fragmentation could impede membrane transport toward the plasma membrane, which affects the turnover of important membrane proteins of dopaminergic neurons such as dopamine transporter protein (DAT), thereby facilitating the progression of PD [106]. Recently, Yi et al. found that Atg9, which is localized to the trans-Golgi network, autophagosomes, and lysosomes in adult *Drosophila* brain glial cells, could regulate the autophagy function of glial. The deletion of glial Atg9 has been observed to induce a progressive loss of DA neurons and locomotion deficits, both of which are characteristic hallmarks of PD. Furthermore, the deletion of glial Atg9 has been observed to induce glial activation, increased release of inflammatory cytokines and ROS production, thereby accelerating DA neurodegeneration [107]. Additionally, Golgi fragmentation has also been observed in other neurodegenerative diseases, such as amyotrophic lateral sclerosis (ALS) and Huntington’s disease (HD) [108–112].

Overall, in the pathogenesis of some neurodegenerative diseases, Golgi fragmentation is exacerbated by the accumulation of pathologic proteins (such as Aβ, tau, and α-synuclein) or intracellular traffic malfunctions, further accelerating the disease progression. And Golgiphagy may serve as an essential mechanism to eliminate damaged Golgi apparatus and thus maintain normal neuronal cell function. It is therefore reasonable to hypothesize that defective Golgiphagy may be a pathological feature of certain neurodegenerative diseases and that the activation of Golgiphagy may mitigate the deleterious effects of the accumulation of pathological proteins, slowing down the disease progression. However, no relevant studies are currently available, and further studies would be required to elucidate the relationship between Golgiphagy and the pathogenesis of these neurodegenerative diseases.

At present, some autophagy activators, such as the Beclin1 activator Isorhynchophyllin [113], have been demonstrated to exert neuroprotective effects in preclinical studies. However, there is a deficit in research targeting Golgiphagy. Modulating the level of Golgiphagy by targeting specific molecules involved in the process, such as the Golgiphagy receptors or other key regulatory proteins, has significant potential for the treatment of neurodegenerative diseases. However, this would require further in-depth studies on the mechanism of Golgiphagy. In conclusion, we believe that Golgiphagy-mediated clearance of dysfunctional Golgi appears to be a promising therapeutic target for the intervention of neurodegenerative diseases.

### Cancer

Abnormalities in Golgi structure and function are closely associated with the progression and metastasis of many types of cancer. The presence of Golgi fragments was first identified in cancer through electron microscopy 50 years ago [17]. Currently, abnormal Golgi structure can be observed in various types of cancer, including prostate [114], colon [27], breast [115], liver [116], and stomach cancers [117]. Abnormalities in the Golgi structure may be caused by the cancer itself. However, Golgi fragmentation can also contribute to the development of cancer through aberrant glycosylation and inhibition of apoptosis in tumor cells. Abnormal glycosylation is a hallmark of cancer [118]. Golgi fragmentation can lead to a disturbed distribution of Golgi-resident glycosyltransferases, resulting in cancer-associated glycosylation defects [119]. During malignant transformation and tumor progression, dispersion of the Golgi can prevent pro-apoptotic kinases from reaching the Golgi apparatus, thereby promoting tumor cell survival and proliferation [120]. GOLPH3, also known as a Golgi-resident oncoprotein, is up-regulated in a variety of cancers and is associated with a poor prognosis. Additionally, as mentioned above, GOLPH3 plays an important role in Golgi fragmentation induced by DNA damage. The depletion of GOLPH3 has been demonstrated to prevent Golgi dispersion and to increase apoptosis. The pathway involving DNA-PK, GOLPH3, and MYO18A is crucial for the survival of tumor cells following DNA damage [11]. This indicates that Golgi fragmentation may play a role in the development of tumors.

There are drugs available that can suppress tumor growth or metastasis by disrupting the Golgi apparatus (Table 1). For example, Brefeldin A has been shown to inhibit tumor growth in vivo [121], but its clinical application has been limited due to its neurotoxicity and low bioavailability. Swainsonine, a Golgi α-mannosidase II inhibitor, has demonstrated promising antitumor activity in gastric cancer and glioma [122, 123], but failed to show efficacy in clinical trials for renal cancer [124]. Consequently, the use of Golgi disruptors may result in the initiation of apoptosis, which could lead to the suppression of tumors. However, there is also a possibility that these agents may exacerbate Golgi fragmentation, thereby increasing the survival of cancer cells.

In contrast, given that autophagy has been demonstrated to facilitate tumor growth in advanced stages of cancer and to enhance drug resistance to a multitude of cancer therapies [125, 126], a plethora of autophagy inhibitors have been developed with the objective of enhancing the efficacy of advanced cancer therapies. Among these, chloroquine (CQ) and its derivative hydroxychloroquine (HCQ) are exemplary autophagy inhibitors that have been subjected to extensive investigation in clinical trials. Mauthe et al. demonstrated that the mechanism of action of CQ may be to inhibit autophagy by impeding the fusion of autophagosomes with lysosomes, and also to induce a profound disorganization of the Golgi and endosomal systems, which may be responsible for the fusion injury [127]. The combination of CQ or HCQ with other chemotherapeutic agents to enhance the efficacy of anticancer therapies has been observed in multiple clinical trials. For instance, in a phase I/II trial of HCQ and gemcitabine in combination prior to surgery, patients exhibited good tolerability and demonstrated a significant reduction in the pancreatic cancer biomarker CA19-9 in 61% of patients at the time of surgery [128]. Furthermore, the combination of HCQ with carboplatin, paclitaxel (and bevacizumab, if criteria are met) in patients with metastatic non-small cell lung cancer demonstrated an objective remission rate of 33% in 30 patients (phase II) and a modest improvement in clinical response [129]. Moreover, a series of small molecule autophagy inhibitors targeting different autophagy stages have been developed, including ULK1 inhibitors, VPS34 inhibitors, V-ATPase inhibitors, lysosomotropic agents and so on [130]. However, the majority of these small molecule inhibitors have yet to be tested in clinical trials, potentially due to their lack of selectivity and unfavorable pharmacological properties. In conclusion, autophagy inhibition has been demonstrated to be a promising therapeutic approach, enhancing the efficacy of chemotherapeutic agents and overcoming drug resistance. Nevertheless, numerous challenges and issues persist, underscoring the urgent need to develop novel autophagy inhibitors that are more specific and efficacious for the cancer.

Recently, Liang et al. developed a new Golgi-targeted platinum (II) complex, Pt3. The researchers showed that Pt3 could specifically target and induce Golgi stress, resulting in Golgi structural rupture, down-regulation of Golgi proteins (GM130, GRASP65/55), Golgi-dependent transport, and loss of glycosylation. This study found that Pt3 triggered Golgiphagy, but inhibited the fusion of autophagosomes with lysosomes during a later stage of autophagy. This modulation of autophagy-apoptosis crosstalk resulted in the killing of cancer cells. The authors also validated the anti-tumor effects of Pt3 in the Lewis lung cancer (LLC) mouse model [131].

Therefore, a novel precision medicine strategy targeting Golgiphagy holds immense significance in advancing anti-tumor therapies.

**Table 1:** Small molecules that target the golgi for tumor inhibition

Drug	Mechanism	Tumor type	References
Brefeldin A	Inhibition of GBF1 and BIGs	colorectal cancer, prostatic cancer, adenoid cystic carcinoma	[18, 121, 132, 133]
AMF-26/M-COPA	Inhibition of GBF1	breast cancer	[134]
Golgicide A	Inhibition of GBF1	breast cancer	[135, 136]
Swainsonine	Inhibition of Golgi mannosidase II	gastric cancer, glioma	[122, 123, 137]
Mannotatin A	Inhibition of Golgi mannosidase II	Melanoma	[138, 139]
IN-9	Inhibition of PI4KIIIβ	lung cancer	[140, 1]
LM11	Inhibition of ARF1 activation through targeting the ARF1-GDP/ARNO complex	breast cancer	[142, 143]

### Conclusions and perspectives

The influence of Golgihagy is an important but currently neglected research direction. Although there is limited evidence, existing studies have demonstrated that Golgiphagy can provide nutrients to cells during starvation and eliminate damaged Golgi. The role of Golgiphagy in neuronal differentiation in vitro has been preliminarily confirmed, and further studies are needed to discover new physiological pathways dependent on Golgiphagy.

At present, given that several Golgiphagy receptors have been identified, it is expected that more novel Golgiphagy receptors and related regulators will be discovered in the future. Nevertheless, it must be acknowledged that our understanding of the molecular mechanisms underlying Golgiphagy remains limited. Additionally, the specific physiological and pathological signals that activate Golgiphagy are yet to be fully elucidated, representing a significant challenge for future research. Furthermore, there are numerous urgent inquiries pertaining to Golgiphagy. For example, is Golgiphagy activated under normal physiological conditions, such as mitosis, when the Golgi apparatus also breaks down into vesicles? What other conditions could also induce Golgiphagy? Are the Golgi membranes that are engulfed by autophagosomes distinct from the Golgi membranes required for the formation of autophagosomes? Does Golgiphagy serve as a quality control mechanism for the Golgi apparatus? How do other pathways, such as the ubiquitin-proteasome pathway, collaborate with Golgiphagy to maintain Golgi homeostasis? Another important point to note in the study of Golgiphagy is that the Golgi, as one of the membrane sources of autophagosomes, plays a crucial role in their formation. Therefore, it is worth investigating whether applying pressure to the entire Golgi will disrupt the autophagic process, and whether damaging only a small portion of the Golgi will result in different responses.

The role of Golgiphagy in human disease will need to be further investigated. Golgiphagy may be directly or indirectly involved in the progression of neurodegenerative diseases by removing ruptured Golgi. In addition, studies on the potential interactions between Golgi and autophagy may help to better understand the effects of Golgiphagy on tumor development. We believe that more studies will be conducted in the near future to reveal the multiple molecular mechanisms underlying Golgiphagy, which will provide new insights into some Golgi-related pathophysiology and may offer new approaches to develop drugs targeting Golgiphagy for disease treatment and intervention.

## Data Availability

Not applicable.

## Abbreviations

ER: Tndoplasmic reticulum
TGN: Trans-Golgi network
Bif: 1/Endophilin B1-Bax-interacting factor 1
mTOR: Mammalian target of rapamycin
GCC88: Coiled-coil domain containing 88 kDa
CMA: Chaperon-mediated autophagy
HSC70: Heat shock cognate 71-kDa protein
ULK: UNC51-like kinase
AMPK: Adenosine 5’-monophosphate-activated protein kinase
FIP200: Focal adhesion kinase family interacting protein of 200 kD
VAPA: Vesicle Associated Membrane Protein-associated protein A
VAPB: Vesicle Associated Membrane Protein-associated protein B
ATG: Autophagy-related
PI3KC3: The class III phosphatidylinositol-3-kinase
PI3P: Phosphatidylinositol 3-phosphate
WIPI2: WD repeat structural domain phosphatidylinositol-interacting protein 2
VMP1: Vacuole membrane protein 1
TMEM41B: Transmembrane protein 41B
ESCRT: Endosomal sorting complex required for transport
SNAREs: Soluble N-ethylmaleimide-sensitive factor attachment protein receptors
HOPS: Homotypic fusion and vacuole protein sorting
RAB: Ras analog in brain
PI4KIIIβ: Phosphatidylinositol-4-kinase-IIIβ
PI4P: Phosphatidylinositol 4-phosphate
AP: Adaptor protein
GRASP55: Golgi ReAssembly Stacking Protein 55
LAMP2: Lysosomal associated membrane protein 2
RUBCNL: Rubicon like autophagy enhancer
STX17: Syntaxin 17
COP I: Coat Protein Complex I
S6K1: S6 Kinase 1
GOLPH3: Golgi phosphoprotein 3
CALCOCO1: Calcium binding and coiled-coil domain protein 1
GMAP: Golgi microtubule-associated protein
YIPF3: The member 3 of Yip1 domain family
YIPF4: The member 4 of Yip1 domain family
MYO18A: Myosin XVIIIA
NDP52: Nuclear dot protein 52 kDa
TAXBP1: TAX1 binding protein 1
LIR: LC3-interacting region
SKICH: SKIP carboxyl homology
UIR: UDS-interacting region
ZDHHC17: Zinc finger DHHC-type palmitoyltransferase 17
zDABM: ZDHHC-AR-binding motif
AR: Ankyrin repeat
CGN: Cis-Golgi network
ERGIC: ER-Golgi intermediate compartment
CAP: Candidate autophagy protein
CLC2: CLATHRIN LIGHT CHAIN 2
HS: Heat stress
NMIIA/MYH9: Non-muscle myosin II A
IRGM: Immune-related guanosine triphosphatase M protein
GBF1: Golgi brefeldin A resistant guanine nucleotide exchange factor 1
NMIIB/MYH10: Non-muscle myosin II B
GOLGA4: Golgin A4
WAC: WW domain-containing adaptor protein with a coiled-coil region
GOLGA2/GM130: Golgin subfamily A member 2
CNS: Central nervous system
PNS: Peripheral nervous system
AD: Alzheimer’s Disease
Aβ: Amyloidβ-protein
NFT: Neurofibrillary tangle
APP: Amyloid precursor protein
PD: Parkinson’s disease
CQ: Chloroquine
HCQ: Hydroxychloroquine

## References

[1] Bentivoglio M. 1898: the golgi apparatus emerges from nerve cells. Trends Neurosci. 1998;21:195–200. 9610881 10.1016/s0166-2236(98)01229-6

[2] Short B, Barr FA. The golgi apparatus. Curr Biol CB. 2000;10:R583–585. 10985372 10.1016/s0960-9822(00)00644-8

[3] Potelle S, Klein A, Foulquier F. Golgi post-translational modifications and associated diseases. J Inherit Metab Dis. 2015;38:741–51. 25967285 10.1007/s10545-015-9851-7

[4] De Matteis MA, Luini A. Exiting the golgi complex. Nat Rev Mol Cell Biol. 2008;9:273–84. 18354421 10.1038/nrm2378

[5] Wei J-H, Seemann J. Unraveling the golgi ribbon. Traffic Cph Den. 2010;11:1391–400. 10.1111/j.1600-0854.2010.01114.x PMC4221251 21040294

[6] Li J, Ahat E, Wang Y. Golgi structure and function in Health, stress, and diseases. Results Probl Cell Differ. 2019;67:441–85. 31435807 10.1007/978-3-030-23173-6_19 PMC7076563

[7] Puthenveedu MA, Bachert C, Puri S, Lanni F, Linstedt AD. GM130 and GRASP65-dependent lateral cisternal fusion allows uniform golgi-enzyme distribution. Nat Cell Biol. 2006;8:238–48. 16489344 10.1038/ncb1366

[8] Colanzi A, Corda D. Mitosis controls the Golgi and the golgi controls mitosis. Curr Opin Cell Biol. 2007;19:386–93. 17689238 10.1016/j.ceb.2007.06.002

[9] Yadav S, Puri S, Linstedt AD. A primary role for Golgi positioning in directed secretion, cell polarity, and wound healing. Mol Biol Cell. 2009;20:1728–36. 19158377 10.1091/mbc.E08-10-1077 PMC2655245

[10] Bisel B, Wang Y, Wei J-H, Xiang Y, Tang D, Miron-Mendoza M, et al. ERK regulates Golgi and centrosome orientation towards the leading edge through GRASP65. J Cell Biol. 2008;182:837–43. 18762583 10.1083/jcb.200805045 PMC2528584

[11] Farber-Katz SE, Dippold HC, Buschman MD, Peterman MC, Xing M, Noakes CJ, et al. DNA damage triggers golgi dispersal via DNA-PK and GOLPH3. Cell. 2014;156:413–27. 24485452 10.1016/j.cell.2013.12.023 PMC4018323

[12] Machamer CE. The golgi complex in stress and death. Front Neurosci. 2015;9:421. 26594142 10.3389/fnins.2015.00421 PMC4635215

[13] Almeida N, Carrara G, Palmeira CM, Fernandes AS, Parsons M, Smith GL, et al. Stimulation of cell invasion by the Golgi Ion Channel GAAP/TMBIM4 via an H2O2-Dependent mechanism. Redox Biol. 2020;28:101361. 31693977 10.1016/j.redox.2019.101361 PMC6838802

[14] Mukherjee S, Chiu R, Leung S-M, Shields D. Fragmentation of the golgi apparatus: an early apoptotic event independent of the cytoskeleton. Traffic Cph Den. 2007;8:369–78. 10.1111/j.1600-0854.2007.00542.x 17394485

[15] Chen J, Chen ZJ. PtdIns4P on dispersed trans-golgi network mediates NLRP3 inflammasome activation. Nature. 2018;564:71–6. 30487600 10.1038/s41586-018-0761-3 PMC9402428

[16] Meng Y, Luo Q, Chen Q, Zhu Y. A noncanonical autophagy function of ATG9A for golgi integrity and dynamics. Autophagy. 2023;19:1607–8. 36198086 10.1080/15548627.2022.2131244 PMC10240965

[17] Maldonado JE, Brown AL, Bayrd ED, Pease GL. Ultrastructure of the myeloma cell. Cancer. 1966;19:1613–27. 5947959 10.1002/1097-0142(196611)19:11<1613::aid-cncr2820191127>3.0.co;2-q

[18] Fujiwara T, Oda K, Yokota S, Takatsuki A, Ikehara Y. Brefeldin A causes disassembly of the golgi complex and accumulation of secretory proteins in the endoplasmic reticulum. J Biol Chem. 1988;263:18545–52. 3192548

[19] Hase T, Summers PL, Dubois DR. Ultrastructural changes of mouse brain neurons infected with Japanese encephalitis virus. Int J Exp Pathol. 1990;71:493–505. 2169298 PMC2002289

[20] Campadelli G, Brandimarti R, Di Lazzaro C, Ward PL, Roizman B, Torrisi MR. Fragmentation and dispersal of Golgi proteins and redistribution of glycoproteins and glycolipids processed through the Golgi apparatus after infection with herpes simplex virus 1. Proc Natl Acad Sci U S A. 1993;90:2798–802. 8385343 10.1073/pnas.90.7.2798 PMC46183

[21] Lavi E, Wang Q, Stieber A, Chen Y, Weiss S, Gonatas NK. Fragmentation and rearrangement of the golgi apparatus during MHV infection of L-2 cells. Adv Exp Med Biol. 1995;380:103–4. 8830461 10.1007/978-1-4615-1899-0_15

[22] Mourelatos Z, Adler H, Hirano A, Donnenfeld H, Gonatas JO, Gonatas NK. Fragmentation of the golgi apparatus of motor neurons in amyotrophic lateral sclerosis revealed by organelle-specific antibodies. Proc Natl Acad Sci U S A. 1990;87:4393–5. 2349244 10.1073/pnas.87.11.4393 PMC54116

[23] Stieber A, Mourelatos Z, Gonatas NK. In Alzheimer’s disease the golgi apparatus of a population of neurons without neurofibrillary tangles is fragmented and atrophic. Am J Pathol. 1996;148:415–26. 8579105 PMC1861703

[24] Sakurai A, Okamoto K, Fujita Y, Nakazato Y, Wakabayashi K, Takahashi H, et al. Fragmentation of the golgi apparatus of the ballooned neurons in patients with corticobasal degeneration and Creutzfeldt-Jakob disease. Acta Neuropathol (Berl). 2000;100:270–4. 10965796 10.1007/s004010000182

[25] Yoshida T, Kamiya T, Imanaka-Yoshida K, Sakakura T. Low cytoplasmic pH causes fragmentation and dispersal of the golgi apparatus in human hepatoma cells. Int J Exp Pathol. 1999;80:51–7. 10365087 10.1046/j.1365-2613.1999.00097.x PMC2517749

[26] Ayala I, Babià T, Baldassarre M, Pompeo A, Fabra A, Kok JW, et al. Morphological and biochemical analysis of the secretory pathway in melanoma cells with distinct metastatic potential. FEBS Lett. 1999;451:315–20. 10371212 10.1016/s0014-5793(99)00620-1

[27] Kellokumpu S, Sormunen R, Kellokumpu I. Abnormal glycosylation and altered golgi structure in colorectal cancer: dependence on intra-golgi pH. FEBS Lett. 2002;516:217–24. 11959136 10.1016/s0014-5793(02)02535-8

[28] Takahashi Y, Meyerkord CL, Hori T, Runkle K, Fox TE, Kester M, et al. Bif-1 regulates Atg9 trafficking by mediating the fission of Golgi membranes during autophagy. Autophagy. 2011;7:61–73. 21068542 10.4161/auto.7.1.14015 PMC3039731

[29] Gosavi P, Houghton FJ, McMillan PJ, Hanssen E, Gleeson PA. The golgi ribbon in mammalian cells negatively regulates autophagy by modulating mTOR activity. J Cell Sci. 2018;131:jcs211987. 29361552 10.1242/jcs.211987

[30] Lu L-Q, Tang M-Z, Qi Z-H, Huang S-F, He Y-Q, Li D-K, et al. Regulation of the golgi apparatus via GOLPH3-mediated new selective autophagy. Life Sci. 2020;253:117700. 32335164 10.1016/j.lfs.2020.117700

[31] Mizushima N. Autophagy: process and function. Genes Dev. 2007;21:2861–73. 18006683 10.1101/gad.1599207

[32] Mijaljica D, Prescott M, Devenish RJ. Microautophagy in mammalian cells: revisiting a 40-year-old conundrum. Autophagy. 2011;7:673–82. 21646866 10.4161/auto.7.7.14733

[33] Kaushik S, Cuervo AM. The coming of age of chaperone-mediated autophagy. Nat Rev Mol Cell Biol. 2018;19:365–81. 29626215 10.1038/s41580-018-0001-6 PMC6399518

[34] Rubinsztein DC, Shpilka T, Elazar Z. Mechanisms of autophagosome biogenesis. Curr Biol CB. 2012;22:R29–34. 22240478 10.1016/j.cub.2011.11.034

[35] Lu G, Wang Y, Shi Y, Zhang Z, Huang C, He W, et al. Autophagy in health and disease: from molecular mechanisms to therapeutic target. MedComm. 2022;3:e150. 35845350 10.1002/mco2.150 PMC9271889

[36] Weerasekara VK, Panek DJ, Broadbent DG, Mortenson JB, Mathis AD, Logan GN, et al. Metabolic-stress-induced rearrangement of the 14-3-3ζ interactome promotes autophagy via a ULK1- and AMPK-regulated 14-3-3ζ interaction with phosphorylated Atg9. Mol Cell Biol. 2014;34:4379–88. 25266655 10.1128/MCB.00740-14 PMC4248729

[37] Ren X, Nguyen TN, Lam WK, Buffalo CZ, Lazarou M, Yokom AL, et al. Structural basis for ATG9A recruitment to the ULK1 complex in mitophagy initiation. Sci Adv. 2023;9:eadg2997. 36791199 10.1126/sciadv.adg2997 PMC9931213

[38] Hu Y, Reggiori F. Molecular regulation of autophagosome formation. Biochem Soc Trans. 2022;50:55–69. 35076688 10.1042/BST20210819 PMC9022990

[39] Yamamoto H, Zhang S, Mizushima N. Autophagy genes in biology and disease. Nat Rev Genet. 2023;24:382–400. 36635405 10.1038/s41576-022-00562-w PMC9838376

[40] Takahashi Y, He H, Tang Z, Hattori T, Liu Y, Young MM, et al. An autophagy assay reveals the ESCRT-III component CHMP2A as a regulator of phagophore closure. Nat Commun. 2018;9:2855. 30030437 10.1038/s41467-018-05254-w PMC6054611

[41] Zhou F, Wu Z, Zhao M, Murtazina R, Cai J, Zhang A, et al. Rab5-dependent autophagosome closure by ESCRT. J Cell Biol. 2019;218:1908–27. 31010855 10.1083/jcb.201811173 PMC6548130

[42] Aman Y, Schmauck-Medina T, Hansen M, Morimoto RI, Simon AK, Bjedov I, et al. Autophagy in healthy aging and disease. Nat Aging. 2021;1:634–50. 34901876 10.1038/s43587-021-00098-4 PMC8659158

[43] Geng J, Klionsky DJ. The golgi as a potential membrane source for autophagy. Autophagy. 2010;6:950–1. 20729630 10.4161/auto.6.7.13009 PMC3359472

[44] Yang Y, Zheng L, Zheng X, Ge L. Autophagosomal membrane origin and formation. Adv Exp Med Biol. 2021;1208:17–42. 34260019 10.1007/978-981-16-2830-6_2

[45] Geng J, Nair U, Yasumura-Yorimitsu K, Klionsky DJ. Post-golgi sec proteins are required for autophagy in Saccharomyces cerevisiae. Mol Biol Cell. 2010;21:2257–69. 20444978 10.1091/mbc.E09-11-0969 PMC2893989

[46] Yamamoto H, Kakuta S, Watanabe TM, Kitamura A, Sekito T, Kondo-Kakuta C, et al. Atg9 vesicles are an important membrane source during early steps of autophagosome formation. J Cell Biol. 2012;198:219–33. 22826123 10.1083/jcb.201202061 PMC3410421

[47] Judith D, Jefferies HBJ, Boeing S, Frith D, Snijders AP, Tooze SA. ATG9A shapes the forming autophagosome through Arfaptin 2 and phosphatidylinositol 4-kinase IIIβ. J Cell Biol. 2019;218:1634–52. 30917996 10.1083/jcb.201901115 PMC6504893

[48] Zhou C, Ma K, Gao R, Mu C, Chen L, Liu Q, et al. Regulation of mATG9 trafficking by src- and ULK1-mediated phosphorylation in basal and starvation-induced autophagy. Cell Res. 2017;27:184–201. 27934868 10.1038/cr.2016.146 PMC5339848

[49] Mattera R, Park SY, De Pace R, Guardia CM, Bonifacino JS. AP-4 mediates export of ATG9A from the trans-golgi network to promote autophagosome formation. Proc Natl Acad Sci U S A. 2017;114:E10697–706. 29180427 10.1073/pnas.1717327114 PMC5740629

[50] Davies AK, Itzhak DN, Edgar JR, Archuleta TL, Hirst J, Jackson LP, et al. AP-4 vesicles contribute to spatial control of autophagy via RUSC-dependent peripheral delivery of ATG9A. Nat Commun. 2018;9:3958. 30262884 10.1038/s41467-018-06172-7 PMC6160451

[51] Zhang X, Wang L, Lak B, Li J, Jokitalo E, Wang Y. GRASP55 senses glucose deprivation through O-GlcNAcylation to Promote Autophagosome-Lysosome Fusion. Dev Cell. 2018;45:245–e2616. 29689198 10.1016/j.devcel.2018.03.023 PMC8207546

[52] Zhang X, Wang L, Ireland SC, Ahat E, Li J, Bekier ME, et al. GORASP2/GRASP55 collaborates with the PtdIns3K UVRAG complex to facilitate autophagosome-lysosome fusion. Autophagy. 2019;15:1787–800. 30894053 10.1080/15548627.2019.1596480 PMC6735621

[53] Ding X, Jiang X, Tian R, Zhao P, Li L, Wang X, et al. RAB2 regulates the formation of autophagosome and autolysosome in mammalian cells. Autophagy. 2019;15:1774–86. 30957628 10.1080/15548627.2019.1596478 PMC6735470

[54] Ahn H-K, Kang YW, Lim HM, Hwang I, Pai H-S. Physiological functions of the COPI Complex in higher plants. Mol Cells. 2015;38:866–75. 26434491 10.14348/molcells.2015.0115 PMC4625068

[55] Nie J, Ma S, Zhang Y, Yu S, Yang J, Li A, et al. COPI Vesicle disruption inhibits mineralization via mTORC1-Mediated autophagy. Int J Mol Sci. 2023;25:339. 38203512 10.3390/ijms25010339 PMC10779376

[56] Wu CC, Taylor RS, Lane DR, Ladinsky MS, Weisz JA, Howell KE. GMx33: a novel family of trans-golgi proteins identified by proteomics. Traffic Cph Den. 2000;1:963–75. 11208086

[57] Bell AW, Ward MA, Blackstock WP, Freeman HN, Choudhary JS, Lewis AP, et al. Proteomics characterization of abundant golgi membrane proteins. J Biol Chem. 2001;276:5152–65. 11042173 10.1074/jbc.M006143200

[58] Snyder CM, Mardones GA, Ladinsky MS, Howell KE. GMx33 associates with the trans-golgi matrix in a dynamic manner and sorts within tubules exiting the Golgi. Mol Biol Cell. 2006;17:511–24. 16236792 10.1091/mbc.E05-07-0682 PMC1345686

[59] Dippold HC, Ng MM, Farber-Katz SE, Lee S-K, Kerr ML, Peterman MC, et al. GOLPH3 bridges phosphatidylinositol-4- phosphate and actomyosin to stretch and shape the Golgi to promote budding. Cell. 2009;139:337–51. 19837035 10.1016/j.cell.2009.07.052 PMC2779841

[60] Ali MF, Chachadi VB, Petrosyan A, Cheng P-W. Golgi phosphoprotein 3 determines cell binding properties under dynamic flow by controlling golgi localization of core 2 N-acetylglucosaminyltransferase 1. J Biol Chem. 2012;287:39564–77. 23027862 10.1074/jbc.M112.346528 PMC3501027

[61] Pereira NA, Pu HX, Goh H, Song Z. Golgi phosphoprotein 3 mediates the golgi localization and function of protein O-linked mannose β-1,2-N-acetlyglucosaminyltransferase 1. J Biol Chem. 2014;289:14762–70. 24733390 10.1074/jbc.M114.548305 PMC4031531

[62] Isaji T, Im S, Gu W, Wang Y, Hang Q, Lu J, et al. An oncogenic protein golgi phosphoprotein 3 up-regulates cell migration via sialylation. J Biol Chem. 2014;289:20694–705. 24895123 10.1074/jbc.M113.542688 PMC4110280

[63] Chen W, Ouyang X, Chen L, Li L. Multiple functions of CALCOCO family proteins in selective autophagy. J Cell Physiol. 2022;237:3505–16. 35853167 10.1002/jcp.30836

[64] Nthiga TM, Kumar Shrestha B, Sjøttem E, Bruun J-A, Bowitz Larsen K, Bhujabal Z, et al. CALCOCO1 acts with VAMP-associated proteins to mediate ER-phagy. EMBO J. 2020;39:e103649. 32525583 10.15252/embj.2019103649 PMC7396842

[65] Stefely JA, Zhang Y, Freiberger EC, Kwiecien NW, Thomas HE, Davis AM, et al. Mass spectrometry proteomics reveals a function for mammalian CALCOCO1 in MTOR-regulated selective autophagy. Autophagy. 2020;16:2219–37. 31971854 10.1080/15548627.2020.1719746 PMC7751563

[66] Nthiga TM, Shrestha BK, Bruun J-A, Larsen KB, Lamark T, Johansen T. Regulation of golgi turnover by CALCOCO1-mediated selective autophagy. J Cell Biol. 2021;220:e202006128. 33871553 10.1083/jcb.202006128 PMC8059076

[67] Barr FA, Short B. Golgins in the structure and dynamics of the golgi apparatus. Curr Opin Cell Biol. 2003;15:405–13. 12892780 10.1016/s0955-0674(03)00054-1

[68] Infante C, Ramos-Morales F, Fedriani C, Bornens M, Rios RM. GMAP-210, a cis-golgi network-associated protein, is a minus end microtubule-binding protein. J Cell Biol. 1999;145:83–98. 10189370 10.1083/jcb.145.1.83 PMC2148210

[69] Pernet-Gallay K, Antony C, Johannes L, Bornens M, Goud B, Rios RM. The overexpression of GMAP-210 blocks anterograde and retrograde transport between the ER and the golgi apparatus. Traffic Cph Den. 2002;3:822–32. 10.1034/j.1600-0854.2002.31107.x 12383348

[70] Sato K, Roboti P, Mironov AA, Lowe M. Coupling of vesicle tethering and Rab binding is required for in vivo functionality of the golgin GMAP-210. Mol Biol Cell. 2015;26:537–53. 25473115 10.1091/mbc.E14-10-1450 PMC4310744

[71] Ríos RM, Sanchís A, Tassin AM, Fedriani C, Bornens M. GMAP-210 recruits gamma-tubulin complexes to cis-golgi membranes and is required for golgi ribbon formation. Cell. 2004;118:323–35. 15294158 10.1016/j.cell.2004.07.012

[72] Smits P, Bolton AD, Funari V, Hong M, Boyden ED, Lu L, et al. Lethal skeletal dysplasia in mice and humans lacking the golgin GMAP-210. N Engl J Med. 2010;362:206–16. 20089971 10.1056/NEJMoa0900158 PMC3108191

[73] Friggi-Grelin F, Rabouille C, Therond P. The cis-golgi Drosophila GMAP has a role in anterograde transport and Golgi organization in vivo, similar to its mammalian ortholog in tissue culture cells. Eur J Cell Biol. 2006;85:1155–66. 16904228 10.1016/j.ejcb.2006.07.001

[74] Rahman A, Lőrincz P, Gohel R, Nagy A, Csordás G, Zhang Y, et al. GMAP is an Atg8a-interacting protein that regulates golgi turnover in Drosophila. Cell Rep. 2022;39:110903. 35649355 10.1016/j.celrep.2022.110903 PMC9637997

[75] Jipa A, Vedelek V, Merényi Z, Ürmösi A, Takáts S, Kovács AL, et al. Analysis of Drosophila Atg8 proteins reveals multiple lipidation-independent roles. Autophagy. 2021;17:2565–75. 33249988 10.1080/15548627.2020.1856494 PMC8496532

[76] Hickey KL, Swarup S, Smith IR, Paoli JC, Miguel Whelan E, Paulo JA, et al. Proteome census upon nutrient stress reveals Golgiphagy membrane receptors. Nature. 2023;623:167–74. 37757899 10.1038/s41586-023-06657-6 PMC10620096

[77] Yang X, Matern HT, Gallwitz D. Specific binding to a novel and essential golgi membrane protein (Yip1p) functionally links the transport GTPases Ypt1p and Ypt31p. EMBO J. 1998;17:4954–63. 9724632 10.1093/emboj/17.17.4954 PMC1170824

[78] Kranjc T, Dempsey E, Cagney G, Nakamura N, Shields DC, Simpson JC. Functional characterisation of the YIPF protein family in mammalian cells. Histochem Cell Biol. 2017;147:439–51. 27999994 10.1007/s00418-016-1527-3

[79] Tanimoto K, Suzuki K, Jokitalo E, Sakai N, Sakaguchi T, Tamura D, et al. Characterization of YIPF3 and YIPF4, cis-Golgi Localizing Yip domain family proteins. Cell Struct Funct. 2011;36:171–85. 21757827 10.1247/csf.11002

[80] Ordureau A, Kraus F, Zhang J, An H, Park S, Ahfeldt T, et al. Temporal proteomics during neurogenesis reveals large-scale proteome and organelle remodeling via selective autophagy. Mol Cell. 2021;81:5082–e509811. 34699746 10.1016/j.molcel.2021.10.001 PMC8688335

[81] Zhou J, Ma J, Yang C, Zhu X, Li J, Zheng X, et al. A non-canonical role of ATG8 in Golgi recovery from heat stress in plants. Nat Plants. 2023;9:749–65. 37081290 10.1038/s41477-023-01398-w

[82] He C, Klionsky DJ. Regulation mechanisms and signaling pathways of Autophagy. Annu Rev Genet. 2009;43:67–93. 19653858 10.1146/annurev-genet-102808-114910 PMC2831538

[83] Petrosyan A, Casey CA, Cheng P-W. The role of Rab6a and phosphorylation of non-muscle myosin IIA tailpiece in alcohol-induced golgi disorganization. Sci Rep. 2016;6:31962. 27535804 10.1038/srep31962 PMC4989220

[84] Macke AJ, Divita TE, Pachikov AN, Mahalingam S, Bellamkonda R, Rasineni K et al. Alcohol-induced Golgiphagy is triggered by the downregulation of golgi GTPase RAB3D. Autophagy. 2024;1–22. 10.1080/15548627.2024.2329476 PMC11210917 38591519

[85] Hansen MD, Johnsen IB, Stiberg KA, Sherstova T, Wakita T, Richard GM, et al. Hepatitis C virus triggers Golgi fragmentation and autophagy through the immunity-related GTPase M. Proc Natl Acad Sci U S A. 2017;114:E3462–71. 28389568 10.1073/pnas.1616683114 PMC5410803

[86] Klabunde B, Wesener A, Bertrams W, Ringshandl S, Halder LD, Vollmeister E, et al. Streptococcus pneumoniae disrupts the structure of the golgi apparatus and subsequent epithelial cytokine response in an H2O2-dependent manner. Cell Commun Signal CCS. 2023;21:208. 37592354 10.1186/s12964-023-01233-x PMC10436572

[87] Aistleitner K, Clark T, Dooley C, Hackstadt T. Selective fragmentation of the trans-golgi apparatus by Rickettsia rickettsii. PLoS Pathog. 2020;16:e1008582. 32421751 10.1371/journal.ppat.1008582 PMC7259798

[88] Nozawa T, Iibushi J, Toh H, Minowa-Nozawa A, Murase K, Aikawa C, et al. Intracellular Group A Streptococcus induces golgi fragmentation to impair host defenses through streptolysin O and NAD-Glycohydrolase. mBio. 2021;12:e01974–20. 33563838 10.1128/mBio.01974-20 PMC7885101

[89] Joachim J, Jefferies HBJ, Razi M, Frith D, Snijders AP, Chakravarty P, et al. Activation of ULK kinase and autophagy by GABARAP trafficking from the centrosome is regulated by WAC and GM130. Mol Cell. 2015;60:899–913. 26687599 10.1016/j.molcel.2015.11.018 PMC4691241

[90] Puri C, Vicinanza M, Ashkenazi A, Gratian MJ, Zhang Q, Bento CF, et al. The RAB11A-Positive compartment is a primary platform for Autophagosome Assembly mediated by WIPI2 recognition of PI3P-RAB11A. Dev Cell. 2018;45:114–e1318. 29634932 10.1016/j.devcel.2018.03.008 PMC5896254

[91] Polson HEJ, de Lartigue J, Rigden DJ, Reedijk M, Urbé S, Clague MJ, et al. Mammalian Atg18 (WIPI2) localizes to omegasome-anchored phagophores and positively regulates LC3 lipidation. Autophagy. 2010;6:506–22. 20505359 10.4161/auto.6.4.11863

[92] Wilson DM, Cookson MR, Van Den Bosch L, Zetterberg H, Holtzman DM, Dewachter I. Hallmarks of neurodegenerative diseases. Cell. 2023;186:693–714. 36803602 10.1016/j.cell.2022.12.032

[93] Tiwari S, Atluri V, Kaushik A, Yndart A, Nair M. Alzheimer’s disease: pathogenesis, diagnostics, and therapeutics. Int J Nanomed. 2019;14:5541–54. 10.2147/IJN.S200490 PMC6650620 31410002

[94] Vassar R, Bennett BD, Babu-Khan S, Kahn S, Mendiaz EA, Denis P, et al. Beta-secretase cleavage of Alzheimer’s amyloid precursor protein by the transmembrane aspartic protease BACE. Science. 1999;286:735–41. 10531052 10.1126/science.286.5440.735

[95] Wang D, Wang L, Zhou Y, Zhao X, Xiong H. The involvement of hematopoietic pre-B cell leukemia transcription factor-interacting protein in regulating epithelial-mesenchymal transition of human spinal glioblastoma. Tumour Biol J Int Soc Oncodevelopmental Biol Med. 2016;37:5897–903. 10.1007/s13277-015-4453-4 26590606

[96] Joshi G, Chi Y, Huang Z, Wang Y. Aβ-induced golgi fragmentation in Alzheimer’s disease enhances Aβ production. Proc Natl Acad Sci U S A. 2014;111:E1230–1239. 24639524 10.1073/pnas.1320192111 PMC3977293

[97] Joshi G, Wang Y. Golgi defects enhance APP amyloidogenic processing in Alzheimer’s disease. BioEssays News Rev Mol Cell Dev Biol. 2015;37:240–7. 10.1002/bies.201400116 PMC4407201 25546412

[98] Jiang Q, Wang L, Guan Y, Xu H, Niu Y, Han L, et al. Golgin-84-associated golgi fragmentation triggers tau hyperphosphorylation by activation of cyclin-dependent kinase-5 and extracellular signal-regulated kinase. Neurobiol Aging. 2014;35:1352–63. 24368089 10.1016/j.neurobiolaging.2013.11.022

[99] de Lau LML, Breteler MMB. Epidemiology of Parkinson’s disease. Lancet Neurol. 2006;5:525–35. 16713924 10.1016/S1474-4422(06)70471-9

[100] Dickson DW. Neuropathology of Parkinson disease. Parkinsonism Relat Disord. 2018;46(Suppl 1):S30–3. 28780180 10.1016/j.parkreldis.2017.07.033 PMC5718208

[101] Fujita Y, Ohama E, Takatama M, Al-Sarraj S, Okamoto K. Fragmentation of Golgi apparatus of nigral neurons with alpha-synuclein-positive inclusions in patients with Parkinson’s disease. Acta Neuropathol (Berl). 2006;112:261–5. 16855830 10.1007/s00401-006-0114-4

[102] Cara-Esteban M, Marín MP, Martínez-Alonso E, Martínez-Bellver S, Teruel-Martí V, Martínez-Menárguez JA, et al. The golgi complex of dopaminergic enteric neurons is fragmented in a hemiparkinsonian rat model. Microsc Res Tech. 2024;87:373–86. 37855309 10.1002/jemt.24442

[103] Tomás M, Martínez-Alonso E, Martínez-Martínez N, Cara-Esteban M, Martínez-Menárguez JA. Fragmentation of the golgi complex of dopaminergic neurons in human substantia nigra: New cytopathological findings in Parkinson’s disease. Histol Histopathol. 2021;36:47–60. 33078843 10.14670/HH-18-270

[104] Lashuel HA, Hirling H. Rescuing defective vesicular trafficking protects against alpha-synuclein toxicity in cellular and animal models of Parkinson’s disease. ACS Chem Biol. 2006;1:420–4. 17168518 10.1021/cb600331e

[105] Rendón WO, Martínez-Alonso E, Tomás M, Martínez-Martínez N, Martínez-Menárguez JA. Golgi fragmentation is Rab and SNARE dependent in cellular models of Parkinson’s disease. Histochem Cell Biol. 2013;139:671–84. 23212845 10.1007/s00418-012-1059-4

[106] Diaz-Corrales FJ, Miyazaki I, Asanuma M, Ruano D, Rios RM. Centrosomal aggregates and Golgi fragmentation disrupt vesicular trafficking of DAT. Neurobiol Aging. 2012;33:2462–77. 22177721 10.1016/j.neurobiolaging.2011.11.014

[107] Yi S, Wang L, Ho MS, Zhang S. The autophagy protein Atg9 functions in glia and contributes to parkinsonian symptoms in a Drosophila model of Parkinson’s disease. Neural Regen Res. 2024;19:1150–5. 37862221 10.4103/1673-5374.382259 PMC10749615

[108] Mourelatos Z, Gonatas NK, Stieber A, Gurney ME, Dal Canto MC. The golgi apparatus of spinal cord motor neurons in transgenic mice expressing mutant Cu,Zn superoxide dismutase becomes fragmented in early, preclinical stages of the disease. Proc Natl Acad Sci U S A. 1996;93:5472–7. 8643599 10.1073/pnas.93.11.5472 PMC39270

[109] van Dis V, Kuijpers M, Haasdijk ED, Teuling E, Oakes SA, Hoogenraad CC, et al. Golgi fragmentation precedes neuromuscular denervation and is associated with endosome abnormalities in SOD1-ALS mouse motor neurons. Acta Neuropathol Commun. 2014;2:38. 24708899 10.1186/2051-5960-2-38 PMC4023628

[110] Sundaramoorthy V, Sultana JM, Atkin JD. Golgi fragmentation in amyotrophic lateral sclerosis, an overview of possible triggers and consequences. Front Neurosci. 2015;9:400. 26578862 10.3389/fnins.2015.00400 PMC4621950

[111] Soo KY, Halloran M, Sundaramoorthy V, Parakh S, Toth RP, Southam KA, et al. Rab1-dependent ER-Golgi transport dysfunction is a common pathogenic mechanism in SOD1, TDP-43 and FUS-associated ALS. Acta Neuropathol (Berl). 2015;130:679–97. 26298469 10.1007/s00401-015-1468-2

[112] Sbodio JI, Snyder SH, Paul BD. Golgi stress response reprograms cysteine metabolism to confer cytoprotection in Huntington’s disease. Proc Natl Acad Sci U S A. 2018;115:780–5. 29317536 10.1073/pnas.1717877115 PMC5789946

[113] Lu J-H, Tan J-Q, Durairajan SSK, Liu L-F, Zhang Z-H, Ma L, et al. Isorhynchophylline, a natural alkaloid, promotes the degradation of alpha-synuclein in neuronal cells via inducing autophagy. Autophagy. 2012;8:98–108. 22113202 10.4161/auto.8.1.18313

[114] Nolfi D, Capone A, Rosati F, Della Giovampaola C. The alpha-1,2 fucosylated tubule system of DU145 prostate cancer cells is derived from a partially fragmented golgi complex and its formation is actin-dependent. Exp Cell Res. 2020;396:112324. 33065114 10.1016/j.yexcr.2020.112324

[115] Sewell R, Bäckström M, Dalziel M, Gschmeissner S, Karlsson H, Noll T, et al. The ST6GalNAc-I sialyltransferase localizes throughout the Golgi and is responsible for the synthesis of the tumor-associated sialyl-Tn O-glycan in human breast cancer. J Biol Chem. 2006;281:3586–94. 16319059 10.1074/jbc.M511826200

[116] Ghadially FN, Parry EW. Ultrastructure of a human hepatocellular carcinoma and surrounding non-neoplastic liver. Cancer. 1966;19:1989–2004. 4289039 10.1002/1097-0142(196612)19:12<1989::aid-cncr2820191226>3.0.co;2-r

[117] He T, Yi Y, Li Y, Xiao Z. [The isolation and assessment of golgi apparatus from gastric cancer cells SGC7901]. Sheng Wu Yi Xue Gong Cheng Xue Za Zhi J Biomed Eng Shengwu Yixue Gongchengxue Zazhi. 2010;27:1085–8. 21089676

[118] Taniguchi N, Kizuka Y. Glycans and cancer: role of N-glycans in cancer biomarker, progression and metastasis, and therapeutics. Adv Cancer Res. 2015;126:11–51. 25727145 10.1016/bs.acr.2014.11.001

[119] Zhang X. Alterations of Golgi Structural Proteins and glycosylation defects in Cancer. Front Cell Dev Biol. 2021;9:665289. 34055798 10.3389/fcell.2021.665289 PMC8149618

[120] Sundram V, Chauhan SC, Jaggi M. Emerging roles of protein kinase D1 in cancer. Mol Cancer Res MCR. 2011;9:985–96. 21680539 10.1158/1541-7786.MCR-10-0365 PMC4030726

[121] Rajamahanty S, Alonzo C, Aynehchi S, Choudhury M, Konno S. Growth inhibition of androgen-responsive prostate cancer cells with brefeldin a targeting cell cycle and androgen receptor. J Biomed Sci. 2010;17:5. 20102617 10.1186/1423-0127-17-5 PMC2843609

[122] Sun J-Y, Zhu M-Z, Wang S-W, Miao S, Xie Y-H, Wang J-B. Inhibition of the growth of human gastric carcinoma in vivo and in vitro by swainsonine. Phytomedicine Int J Phytother Phytopharm. 2007;14:353–9. 10.1016/j.phymed.2006.08.003 17097281

[123] Sun J-Y, Yang H, Miao S, Li J-P, Wang S-W, Zhu M-Z, et al. Suppressive effects of swainsonine on C6 glioma cell in vitro and in vivo. Phytomedicine Int J Phytother Phytopharm. 2009;16:1070–4. 10.1016/j.phymed.2009.02.012 19427771

[124] Shaheen PE, Stadler W, Elson P, Knox J, Winquist E, Bukowski RM. Phase II study of the efficacy and safety of oral GD0039 in patients with locally advanced or metastatic renal cell carcinoma. Invest New Drugs. 2005;23:577–81. 16034517 10.1007/s10637-005-0793-z

[125] Mulcahy Levy JM, Zahedi S, Griesinger AM, Morin A, Davies KD, Aisner DL, et al. Autophagy inhibition overcomes multiple mechanisms of resistance to BRAF inhibition in brain tumors. eLife. 2017;6:e19671. 28094001 10.7554/eLife.19671 PMC5241115

[126] Mele L, Del Vecchio V, Liccardo D, Prisco C, Schwerdtfeger M, Robinson N, et al. The role of autophagy in resistance to targeted therapies. Cancer Treat Rev. 2020;88:102043. 32505806 10.1016/j.ctrv.2020.102043

[127] Mauthe M, Orhon I, Rocchi C, Zhou X, Luhr M, Hijlkema K-J, et al. Chloroquine inhibits autophagic flux by decreasing autophagosome-lysosome fusion. Autophagy. 2018;14:1435–55. 29940786 10.1080/15548627.2018.1474314 PMC6103682

[128] Boone BA, Bahary N, Zureikat AH, Moser AJ, Normolle DP, Wu W-C, et al. Safety and Biologic Response of pre-operative autophagy inhibition in combination with Gemcitabine in patients with pancreatic adenocarcinoma. Ann Surg Oncol. 2015;22:4402–10. 25905586 10.1245/s10434-015-4566-4 PMC4663459

[129] Malhotra J, Jabbour S, Orlick M, Riedlinger G, Guo Y, White E, et al. Phase Ib/II study of hydroxychloroquine in combination with chemotherapy in patients with metastatic non-small cell lung cancer (NSCLC). Cancer Treat Res Commun. 2019;21:100158. 31521049 10.1016/j.ctarc.2019.100158

[130] Chen J-L, Wu X, Yin D, Jia X-H, Chen X, Gu Z-Y, et al. Autophagy inhibitors for cancer therapy: small molecules and nanomedicines. Pharmacol Ther. 2023;249:108485. 37406740 10.1016/j.pharmthera.2023.108485

[131] Liang B-B, Liu Q, Liu B, Yao H-G, He J, Tang C-F, et al. A golgi-targeted platinum Complex plays a dual role in Autophagy Regulation for highly efficient Cancer therapy. Angew Chem Int Ed Engl. 2023;62:e202312170. 37710398 10.1002/anie.202312170

[132] Zhou L, Gao W, Wang K, Huang Z, Zhang L, Zhang Z, et al. Brefeldin A inhibits colorectal cancer growth by triggering Bip/Akt-regulated autophagy. FASEB J off Publ Fed Am Soc Exp Biol. 2019;33:5520–34. 10.1096/fj.201801983R 30668917

[133] Salles FT, Hespanhol AM, Jaeger RG, Marques MM. Brefeldin-A induces apoptosis in human adenoid cystic carcinoma cultured cells. Oral Oncol. 2004;40:585–90. 15063386 10.1016/j.oraloncology.2003.12.007

[134] Ohashi Y, Iijima H, Yamaotsu N, Yamazaki K, Sato S, Okamura M, et al. AMF-26, a novel inhibitor of the golgi system, targeting ADP-ribosylation factor 1 (Arf1) with potential for cancer therapy. J Biol Chem. 2012;287:3885–97. 22158626 10.1074/jbc.M111.316125 PMC3281721

[135] Sáenz JB, Sun WJ, Chang JW, Li J, Bursulaya B, Gray NS, et al. Golgicide A reveals essential roles for GBF1 in Golgi assembly and function. Nat Chem Biol. 2009;5:157–65. 19182783 10.1038/nchembio.144 PMC3500152

[136] Luchsinger C, Aguilar M, Burgos PV, Ehrenfeld P, Mardones GA. Functional disruption of the golgi apparatus protein ARF1 sensitizes MDA-MB-231 breast cancer cells to the antitumor drugs actinomycin D and vinblastine through ERK and AKT signaling. PLoS ONE. 2018;13:e0195401. 29614107 10.1371/journal.pone.0195401 PMC5882166

[137] Tulsiani DR, Harris TM, Touster O. Swainsonine inhibits the biosynthesis of complex glycoproteins by inhibition of golgi mannosidase II. J Biol Chem. 1982;257:7936–9. 6806288

[138] Tropea JE, Kaushal GP, Pastuszak I, Mitchell M, Aoyagi T, Molyneux RJ, et al. Mannostatin A, a new glycoprotein-processing inhibitor. Biochemistry. 1990;29:10062–9. 2271638 10.1021/bi00495a008

[139] Ochi Y, Atsumi S, Aoyagi T, Umezawa K. Inhibition of tumor cell invasion in the Boyden chamber assay by a mannosidase inhibitor, mannostatin A. Anticancer Res. 1993;13:1421–4. 8239514

[140] Rutaganira FU, Fowler ML, McPhail JA, Gelman MA, Nguyen K, Xiong A, et al. Design and structural characterization of potent and selective inhibitors of Phosphatidylinositol 4 kinase IIIβ. J Med Chem. 2016;59:1830–9. 26885694 10.1021/acs.jmedchem.5b01311 PMC5289284

[141] Tan X, Banerjee P, Pham EA, Rutaganira FUN, Basu K, Bota-Rabassedas N, et al. PI4KIIIβ is a therapeutic target in chromosome 1q-amplified lung adenocarcinoma. Sci Transl Med. 2020;12:eaax3772. 31969487 10.1126/scitranslmed.aax3772 PMC7702266

[142] Viaud J, Zeghouf M, Barelli H, Zeeh J-C, Padilla A, Guibert B, et al. Structure-based discovery of an inhibitor of Arf activation by Sect. 7 domains through targeting of protein-protein complexes. Proc Natl Acad Sci U S A. 2007;104:10370–5. 17563369 10.1073/pnas.0700773104 PMC1965520

[143] Xie X, Tang S-C, Cai Y, Pi W, Deng L, Wu G, et al. Suppression of breast cancer metastasis through the inactivation of ADP-ribosylation factor 1. Oncotarget. 2016;7:58111–20. 27517156 10.18632/oncotarget.11185 PMC5295416

